# Gut microbiota and microbiota-derived metabolites in radiation-induced injury: mechanisms and therapeutic opportunities

**DOI:** 10.3389/fmicb.2026.1923929

**Published:** 2026-09-04

**Authors:** Zhiyan Zou, Huan Liu, Yan Hu, Xinyun Ge, Jie Zhang, Xiaoan Li

**Affiliations:** 1 NHC Key Laboratory of Nuclear Technology Medical Transformation, Mianyang Central Hospital, School of Medicine, University of Electronic Science and Technology of China, Mianyang, China; 2 Department of Pediatrics, Mianyang Central Hospital, School of Medicine, University of Electronic Science and Technology of China, Mianyang, China; 3 Sichuan Provincial Engineering Research Center of Nuclear Medical Equipment Translation and Application, Mianyang Central Hospital, School of Medicine, University of Electronic Science and Technology of China, Mianyang, China; 4 School of Life Science and Engineering, Southwest University of Science and Technology, Mianyang, China

**Keywords:** gut microbiota, microbial metabolites, microbiota-targeted intervention, radiation-induced injury, radioprotection

## Abstract

Radiation-induced injury is a complex pathological process involving DNA damage, oxidative stress, inflammatory amplification, epithelial barrier disruption, immune dysregulation, metabolic remodeling, and impaired tissue repair. Accumulating evidence indicates that the gut microbiota is not merely altered by irradiation but may also modify host radiation responses and tissue recovery; however, direct causal support remains confined to selected microbes, metabolites, and pathways, predominantly in preclinical models. Irradiation is frequently associated with reduced microbial diversity, depletion of selected commensals, expansion of opportunistic pathobionts, and altered microbial metabolic output; selected experimental studies further indicate that these changes can modify intestinal injury and host recovery. Conversely, defined microbes and metabolites have improved radiation outcomes in preclinical intervention models by supporting epithelial, immune, and metabolic homeostasis. In this review, we summarize current advances in understanding the gut microbiota–metabolite axis in radiation-induced injury. We first discuss radiation-induced gut dysbiosis and the major microbial metabolites involved, including short-chain fatty acids, tryptophan-derived metabolites, bile acids, lipid metabolites, polyamines, and other bioactive molecules. We then highlight the key mechanisms by which gut microbiota and microbial metabolites mitigate radiation injury, including suppression of oxidative stress, inhibition of inflammatory signaling, restoration of epithelial barrier integrity, promotion of intestinal stem cell-mediated regeneration, regulation of immune homeostasis, and modulation of lipid peroxidation-associated ferroptosis. Finally, we evaluate microbiota-targeted intervention strategies, including probiotics, prebiotics, postbiotics, fecal microbiota transplantation, antibiotic modulation, natural products, engineered probiotics, and nanomedicine-assisted delivery systems. Overall, the gut microbiota–metabolite axis represents a biologically plausible and increasingly testable target for radioprotection and mitigation, although its clinical utility remains to be established. Future studies should move beyond descriptive microbiome profiling toward causal, function-oriented, and multi-omics-driven investigations, with particular emphasis on the microbiota–lipid metabolism–ferroptosis axis and precision microbiome therapeutics. Carefully validated microbiota-targeted approaches may provide future opportunities to reduce normal-tissue toxicity and improve recovery without compromising tumor control.

## 1 Introduction

Ionizing radiation is widely used in medicine, industry, and scientific research, while accidental and environmental exposure continues to pose significant health risks (Jackson and Bartek, 2009; Hauer-Jensen et al., 2014). In oncology, radiotherapy remains a cornerstone of cancer treatment, yet the unavoidable irradiation of surrounding normal tissues frequently results in acute and chronic toxicities that compromise treatment efficacy and patients' quality of life (Hauer-Jensen et al., 2014; Blake et al., 2024). Depending on radiation dose, exposure pattern, and tissue radiosensitivity, radiation injury can affect multiple organ systems, including the hematopoietic, gastrointestinal (GI), cardiovascular, pulmonary, nervous, and immune systems (Hauer-Jensen et al., 2014; Hanania et al., 2019; Yang et al., 2020). Among these organs, the intestine is particularly susceptible to radiation because of its rapid epithelial turnover and intimate interaction with the gut microbiota (Moraitis et al., 2023; Dong et al., 2022). Radiation disrupts intestinal epithelial regeneration, damages the mucosal barrier, increases intestinal permeability, and promotes local and systemic inflammation (Hauer-Jensen et al., 2014; Wang et al., 2024). Simultaneously, irradiation profoundly alters the composition and function of the gut microbiota, leading to microbial dysbiosis that further aggravates epithelial injury, immune dysfunction, and impaired tissue repair (Wang et al., 2024; Moraitis et al., 2023; Yi et al., 2023; Crawford and Gordon, 2005). Consequently, the intestine functions not only as one of the primary targets of radiation toxicity but also as a central regulator of systemic responses to irradiation through the gut microbiota–host axis (Moraitis et al., 2023; Maddern et al., 2023).

Current strategies for preventing and treating radiation-induced injury mainly rely on supportive care and symptom management, including anti-inflammatory agents, antioxidants, mucosal protectants, hematopoietic growth factors, antibiotics, and nutritional support (Hauer-Jensen et al., 2014; Wang et al., 2024). Although several targeted agents, such as amifostine and palifermin, have demonstrated clinical benefits in specific settings, their application remains limited by restricted indications, adverse effects, or insufficient efficacy across different radiation-induced complications (Hauer-Jensen et al., 2014). Radiation injury is driven by a complex network of interconnected pathological events, including DNA damage (Jackson and Bartek, 2009), oxidative stress (Zhao and Robbins, 2009; Mohammadgholi and Hosseinimehr, 2024), mitochondrial dysfunction (Rong et al., 2025), epithelial and endothelial cell death, immune dysregulation, microbial imbalance (Wang et al., 2024; Crawford and Gordon, 2005), and metabolic disturbances. Consequently, conventional single-target therapies often fail to provide durable protection or promote complete tissue regeneration, particularly in radiation-induced intestinal injury, where epithelial stem cell loss, barrier disruption, chronic inflammation, fibrosis, and gut microbiota dysbiosis coexist (Hauer-Jensen et al., 2014; Wang et al., 2024; Moraitis et al., 2023). These limitations highlight the urgent need for multi-target therapeutic strategies capable of restoring tissue homeostasis while simultaneously regulating host immunity and the intestinal microenvironment.

Growing evidence has established the gut microbiota as an active regulator of radiation responses rather than a passive victim of irradiation (Blake et al., 2024; Wang et al., 2024; Moraitis et al., 2023; Yi et al., 2023; Dong et al., 2022; Crawford and Gordon, 2005; Guo et al., 2020). Ionizing radiation not only disrupts microbial composition and function but also reshapes microbial metabolic activities, thereby influencing host susceptibility to radiation injury, inflammatory responses, tissue repair, and long-term recovery (Crawford and Gordon, 2005; Guo et al., 2020; Li et al., 2020). Conversely, baseline microbial composition and metabolic capacity can affect individual radiosensitivity, suggesting that the gut microbiota may serve as both a predictive biomarker and a therapeutic target for radiation-induced toxicity (Iacovacci et al., 2024; Ma et al., 2025; Yu et al., 2025). Microbiota-derived metabolites constitute the principal molecular interface between intestinal microorganisms and the host. Metabolites such as short-chain fatty acids (Koh et al., 2016; Tan et al., 2014; Parada Venegas et al., 2019), tryptophan metabolites (Zelante et al., 2013; Rosser et al., 2020; Xie et al., 2024), bile acids (Jiao et al., 2025; Cheng et al., 2026), polyamines (Mafe and Büsselberg, 2026), and lipid-related metabolites (Cui et al., 2025) regulate epithelial integrity, immune homeostasis, oxidative stress, and tissue regeneration through diverse host signaling pathways. Recent multi-omics studies integrating metagenomics, metabolomics, transcriptomics, and functional validation have further demonstrated that specific microbial communities and metabolite signatures are closely associated with radiation tolerance, epithelial regeneration, and survival following irradiation (Guo et al., 2020; Li et al., 2020; Lu et al., 2025). These findings indicate that modulation of the gut microbiota–metabolite axis represents a promising strategy for mitigating radiation-induced injury.

Accordingly, microbiota-based interventions—including probiotics, prebiotics, postbiotics, fecal microbiota transplantation (FMT), natural product-derived microbiota modulators, engineered bacteria, and defined metabolite therapies—have attracted increasing attention because of their ability to simultaneously regulate multiple pathogenic processes, restore microbial homeostasis, and enhance host resilience to radiation (Wang et al., 2024; Yi et al., 2023; Maddern et al., 2023; Palkovsky et al., 2025; Lu et al., 2024). Compared with conventional pharmacological approaches, these strategies offer greater potential for system-level and multi-target intervention. In this review, we summarize current advances in understanding the roles of gut microbiota and microbiota-derived metabolites in radiation-induced injury, with particular emphasis on intestinal injury and the underlying molecular mechanisms. We further discuss emerging microbiota-targeted therapeutic strategies, recent progress in multi-omics research, current challenges, and future perspectives for developing next-generation radioprotective interventions.

Importantly, the current evidence supporting the role of the gut microbiota–metabolite axis in radiation-induced injury originates from heterogeneous experimental systems, including animal irradiation models, human observational studies, multi-omics analyses, and limited clinical interventions. Therefore, the interpretation of microbiota-associated findings requires careful consideration of evidence strength and biological context. While animal studies have provided substantial mechanistic insights into microbial regulation of oxidative stress, inflammation, epithelial regeneration, and immune responses, translation of these findings into clinical radioprotection remains incomplete. In this review, we therefore distinguish between associative evidence, mechanistic validation, preclinical therapeutic evidence, and clinical observations to provide a balanced assessment of the current state of microbiota-based radioprotection (Blake et al., 2024; Wang et al., 2024; Guo et al., 2020; Iacovacci et al., 2024; Ma et al., 2025; Yu et al., 2025).

## 2 Biological basis of radiation-induced injury

### 2.1 Direct and indirect effects of ionizing radiation

**Figure 1:**
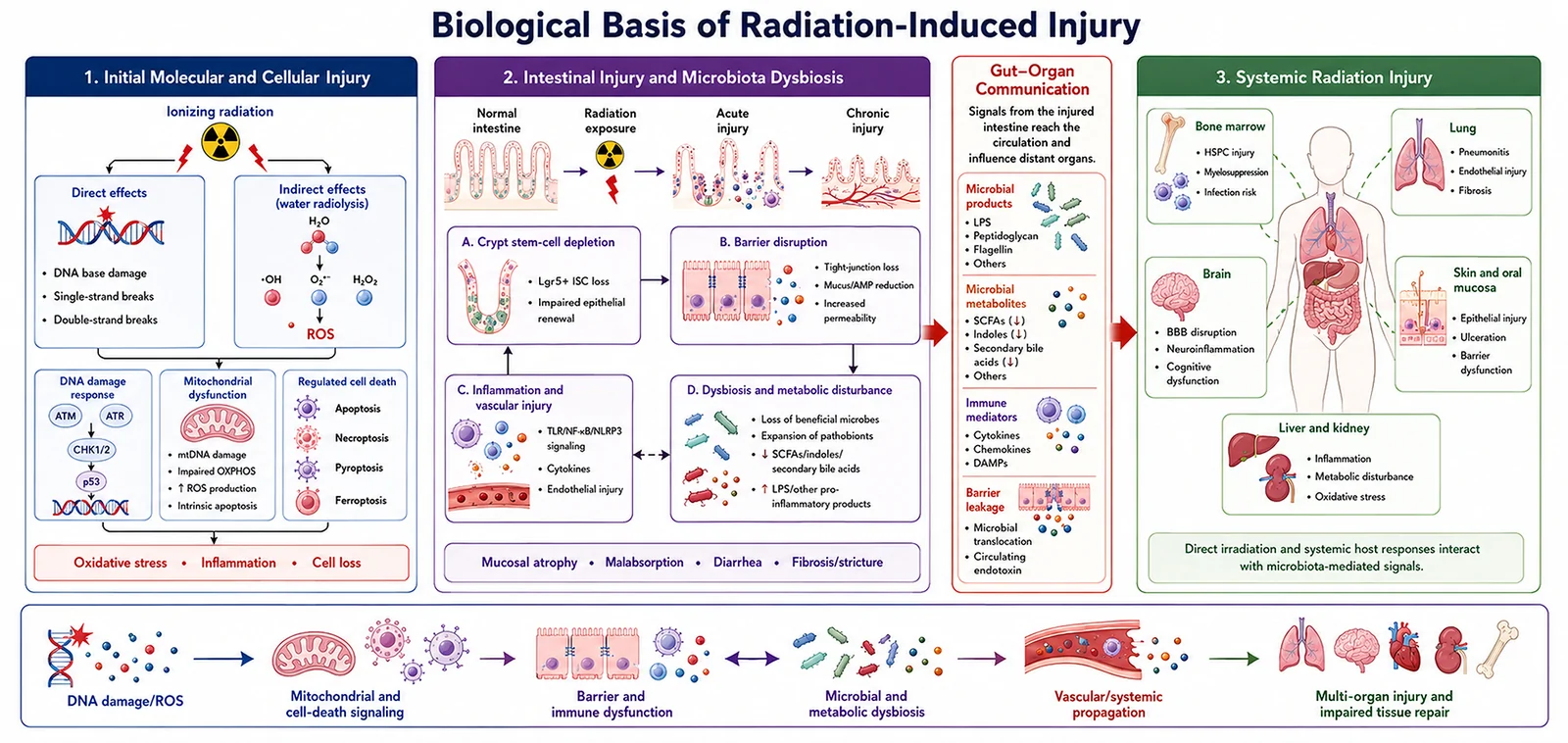
Biological basis of radiation-induced injury from molecular damage to systemic multi-organ dysfunction. Ionizing radiation initiates tissue injury through direct DNA damage and indirect oxidative stress resulting from water radiolysis and reactive oxygen species (ROS) generation. These primary insults activate the DNA damage response (DDR), impair mitochondrial function, and induce regulated cell-death pathways, including apoptosis, necroptosis, pyroptosis, and ferroptosis. The gastrointestinal tract is particularly radiosensitive because of its rapid epithelial turnover and dependence on intestinal stem cells. Radiation-induced gastrointestinal injury involves stem-cell depletion, crypt loss, epithelial barrier disruption, inflammatory and vascular injury, microbial dysbiosis, metabolic disturbance, and impaired mucosal regeneration. Barrier failure further facilitates microbial translocation and systemic exposure to microbial products, metabolites, and immune mediators. Beyond the gut, direct irradiation and systemic host responses interact with microbiota-associated signals to influence injury in the hematopoietic system, lung, brain, skin, oral mucosa, liver, and kidney. The contribution of microbiota-dependent signaling varies across organs and experimental contexts. Collectively, radiation injury represents a dynamic, multiscale process linking molecular damage, cellular stress, epithelial and immune dysfunction, microbiota dysbiosis, gut–organ communication, and impaired tissue repair.

Radiation-induced injury begins with the deposition of ionizing energy in biological tissues (Jackson and Bartek, 2009). At the cellular level, ionizing radiation damages biomolecules through direct and indirect mechanisms. Direct effects occur when radiation energy is deposited directly onto DNA, producing base lesions, single-strand breaks, DNA–protein crosslinks, and double-strand breaks (DSBs) (Jackson and Bartek, 2009; Ward, 1988). Among these lesions, DSBs are the most lethal because inaccurate or incomplete repair can result in chromosomal instability, mutations, mitotic catastrophe, senescence, or cell death (Jackson and Bartek, 2009; Bakkenist and Kastan, 2003). The progression from initial molecular and cellular injury to intestinal dysfunction, microbiota dysbiosis, gut–organ communication, and systemic radiation injury is summarized in Figure 1.

The biological complexity of radiation-induced DNA damage also depends strongly on radiation quality. High linear energy transfer (high-LET) radiation deposits energy densely along particle tracks and therefore produces spatially clustered DNA damage containing multiple lesions—including DSBs, single-strand breaks, oxidized bases, and abasic sites—within one or two helical turns of DNA. Such complex lesions are considerably more difficult to repair accurately than isolated lesions generated predominantly by low-LET radiation and therefore have a greater probability of generating persistent chromosome aberrations, mutations, and genomic instability (Hada and Georgakilas, 2008).

Importantly, radiation-induced damage can persist long after the initial physical energy deposition. Unrepaired or misrepaired DNA lesions may sustain DNA damage response (DDR) signaling, promote chromosome mis-segregation, and generate micronuclei during subsequent mitoses. These processes contribute to delayed genomic instability and may provide cytosolic DNA signals capable of engaging innate immune pathways, although the relationship between micronuclei and cyclic GMP–AMP synthase–stimulator of interferon genes (cGAS–STING) activation appears to be context dependent. Persistent mitochondrial dysfunction further maintains elevated reactive oxygen species (ROS) production, converting the transient physicochemical radiation insult into chronic oxidative stress (Jackson and Bartek, 2009; Zhao and Robbins, 2009; Rong et al., 2025; Bakhoum et al., 2018).

Cells that survive substantial DNA damage may also enter stress-induced premature senescence rather than undergoing immediate cell death. Senescent cells remain metabolically active and develop a senescence-associated secretory phenotype (SASP), characterized by the release of pro-inflammatory cytokines, chemokines, growth factors, and matrix-remodeling enzymes. Accumulation of senescent cells and SASP signaling can propagate inflammation to neighboring cells, impair stem-cell function and tissue regeneration, and contribute to late radiation toxicity and fibrosis. Thus, persistent DDR signaling, genomic instability, oxidative stress, and cellular senescence provide an important mechanistic bridge between the initial radiation event and delayed tissue dysfunction (Zhao and Robbins, 2009; Faget et al., 2019).

Indirect effects are mainly mediated by water radiolysis, which generates ROS, including hydroxyl radicals, superoxide anions, and hydrogen peroxide (Ward, 1988; Zhao and Robbins, 2009). These ROS amplify the initial radiation insult by damaging DNA, membrane lipids, proteins, and organelles (Zhao and Robbins, 2009; Mohammadgholi and Hosseinimehr, 2024). Mitochondria are particularly vulnerable to oxidative injury. Radiation-induced mitochondrial dysfunction impairs oxidative phosphorylation, depletes adenosine triphosphate (ATP), promotes further ROS production, and activates intrinsic apoptotic signaling, thereby sustaining cellular damage beyond the initial exposure (Rong et al., 2025). In response to DNA damage and oxidative stress, cells activate DNA damage response pathways, primarily involving ataxia-telangiectasia mutated (ATM), ATM- and Rad3-related (ATR), checkpoint kinase 1/2 (CHK1/CHK2), and p53 signaling (Jackson and Bartek, 2009; Bakkenist and Kastan, 2003). These pathways induce cell-cycle arrest and facilitate DNA repair through homologous recombination or non-homologous end joining. When damage exceeds repair capacity, cells undergo senescence or death (Jackson and Bartek, 2009). Apoptosis remains the best-characterized form of radiation-induced cell death (Jeffers et al., 2003), but necrosis, pyroptosis, and ferroptosis also contribute to radiation pathology (Dixon et al., 2012; Stockwell et al., 2017; Jiang et al., 2021; Lei et al., 2020; Zhang et al., 2022). Pyroptosis links inflammasome activation to inflammatory cytokine release (Zhang et al., 2022), whereas ferroptosis is driven by iron-dependent lipid peroxidation and antioxidant failure (Dixon et al., 2012; Yang et al., 2014; Stockwell et al., 2017; Jiang et al., 2021; Stockwell, 2022; Kagan et al., 2017; Doll et al., 2017; Lei et al., 2020; Nishizawa et al., 2023).

Thus, radiation injury is not caused by a single molecular event but by an interconnected network involving DNA damage (Jackson and Bartek, 2009; Bakkenist and Kastan, 2003; Ward, 1988), ROS accumulation (Zhao and Robbins, 2009; Mohammadgholi and Hosseinimehr, 2024), mitochondrial dysfunction (Rong et al., 2025), impaired repair, inflammatory cell death (Dixon et al., 2012; Stockwell et al., 2017; Jiang et al., 2021; Lei et al., 2020; Zhang et al., 2022), and tissue-level immune activation. The relative contribution of each process depends on radiation dose, dose rate, tissue type, cellular radiosensitivity, and the local microenvironment. This complexity provides the biological rationale for multi-target radioprotective strategies, including interventions that regulate oxidative stress, inflammation, metabolism, and host–microbiota interactions.

### 2.2 Radiation-induced gastrointestinal injury

The gastrointestinal tract is among the most radiosensitive organs because of its rapid epithelial renewal, dependence on intestinal stem cells (ISCs), complex crypt–villus architecture, abundant immune cells, dense vasculature, and close interaction with the gut microbiota (Hauer-Jensen et al., 2014; Barker et al., 2007). Radiation-induced gastrointestinal injury, especially radiation enteritis, is a major dose-limiting toxicity in abdominal and pelvic radiotherapy and a key component of the gastrointestinal subsyndrome of acute radiation syndrome (Hauer-Jensen et al., 2014; Moraitis et al., 2023). The intestinal epithelium is continuously regenerated by Lgr5^+^ crypt-base columnar stem cells and transit-amplifying progenitors (Barker et al., 2007). Ionizing radiation preferentially damages these highly proliferative cells, leading to DNA damage, p53-dependent apoptosis (Komarova et al., 2004), stem cell depletion, and impaired epithelial regeneration (Qiu et al., 2010). As a result, the production of absorptive enterocytes, goblet cells, paneth cells, and enteroendocrine cells is reduced. Histologically, this is reflected by crypt loss, villus shortening, mucosal thinning, epithelial denudation, and, in chronic stages, fibrosis, vascular injury, and persistent inflammation (Hauer-Jensen et al., 2014).

Barrier dysfunction is a central event in radiation-induced intestinal injury (Hauer-Jensen et al., 2014; Wang et al., 2024). Radiation disrupts tight junction proteins, including zonula occludens-1 (ZO-1), occludin, and claudins, while impairing mucus secretion and antimicrobial peptide production. These changes weaken both the physical and chemical barriers of the intestinal mucosa, increase epithelial permeability, and facilitate the translocation of bacteria, lipopolysaccharide (LPS), and other microbial products into the lamina propria and circulation (Hauer-Jensen et al., 2014; Zhang et al., 2022). Microbial translocation activates innate immune receptors, including Toll-like receptors (TLRs) and nucleotide-binding oligomerization domain (NOD)-like receptors, on epithelial and immune cells. This promotes NF-κB and inflammasome signaling, resulting in the release of pro-inflammatory cytokines, chemokines, and lipid mediators (Zhao and Robbins, 2009; Mohammadgholi and Hosseinimehr, 2024). The resulting inflammatory microenvironment further damages the epithelium, recruits immune cells, delays mucosal repair, and forms a self-amplifying cycle of barrier disruption and inflammation.

Radiation also reshapes the gut microbial ecosystem. Radiation-induced dysbiosis is commonly characterized by reduced microbial diversity, loss of beneficial anaerobic commensals, expansion of opportunistic pathobionts, and altered microbial metabolism (Wang et al., 2024; Crawford and Gordon, 2005; Guo et al., 2020; Li et al., 2020). These changes reduce the production of protective metabolites such as short-chain fatty acids (SCFAs) and indole derivatives while increasing pro-inflammatory microbial products (Guo et al., 2020; Li et al., 2020; Lu et al., 2025). Therefore, dysbiosis can arise as a consequence of tissue injury, while selected transfer, depletion, reconstitution, microbial, and metabolite-intervention studies indicate that specific microbial changes can also modify injury severity to epithelial damage, immune activation, and defective tissue repair (Wang et al., 2024; Moraitis et al., 2023; Yang et al., 2023). Accordingly, radiation enteritis should be viewed as an integrated disorder involving intestinal stem cell loss (Barker et al., 2007; Qiu et al., 2010), oxidative stress (Zhao and Robbins, 2009; Mohammadgholi and Hosseinimehr, 2024), mitochondrial injury (Rong et al., 2025), immune dysregulation, vascular damage, epithelial barrier failure, microbial dysbiosis (Crawford and Gordon, 2005; Guo et al., 2020; Li et al., 2020), and metabolic disturbance. This perspective supports therapeutic approaches that simultaneously restore epithelial integrity, suppress inflammation, and remodel the gut microbiota–metabolite axis.

### 2.3 Systemic radiation injury beyond the gut

Mechanistically, gut–organ communication after irradiation is likely mediated through several overlapping routes. In the gut–bone marrow axis, circulating microbial metabolites can influence hematopoietic stem and progenitor cell metabolism, inflammatory tone, and immune-cell differentiation, whereas disruption of the intestinal barrier can expose hematopoietic tissues to microbial products and systemic inflammatory signals. Experimental evidence is particularly compelling for microbial metabolites such as propionate and tryptophan derivatives, which have been shown to improve hematopoietic recovery after irradiation (Guo et al., 2020; Xie et al., 2024).

The gut–lung axis is mediated by circulating microbial metabolites, microbial products, and gut-educated immune cells. Dysbiosis-associated loss of immunoregulatory metabolites may favor systemic inflammatory responses that amplify pulmonary immune-cell recruitment and fibrotic signaling. Conversely, SCFAs and other microbial metabolites can influence macrophage, neutrophil, and lymphocyte function, providing a plausible mechanism through which intestinal ecology may modify radiation-induced pneumonitis or fibrosis (Chen et al., 2021; Budden et al., 2017).

Within the gut–brain axis, microbial metabolites and systemic cytokines can influence blood–brain barrier integrity, microglial activation, neuroinflammation, and neuroendocrine signaling. Radiation-induced intestinal permeability may further increase systemic exposure to microbial products capable of altering neuroimmune homeostasis. However, compared with the gut–bone marrow axis, direct causal evidence linking specific gut microbes or metabolites to radiation-induced brain or lung injury remains relatively limited. These pathways should therefore be regarded as biologically plausible and increasingly supported inter-organ networks rather than uniformly established mechanisms (Luo et al., 2022; Cryan et al., 2019).

## 3 Radiation-induced gut microbiota dysbiosis

### 3.1 Changes in microbial diversity following irradiation

Radiation-induced gut microbiota dysbiosis is a hallmark of radiation injury, linking epithelial damage with immune dysregulation, metabolic disturbance, and systemic toxicity (Wang et al., 2024; Crawford and Gordon, 2005; Guo et al., 2020). Following ionizing radiation exposure, the gut microbiota frequently exhibits ecological changes characterized by reduced microbial richness, impaired community stability, increased inter-individual variability, and marked alterations in community composition (Li et al., 2020; Lu et al., 2025; Zhang et al., 2023). These changes are commonly assessed using alpha-diversity indices (e.g., Chao1, ACE, Shannon) to evaluate microbial richness and evenness, together with beta-diversity analyses, including principal coordinate analysis (PCoA), non-metric multidimensional scaling (NMDS), and UniFrac-based clustering, to characterize shifts in overall community structure (Li et al., 2020; Lu et al., 2025; Zhang et al., 2023).

The extent and trajectory of microbial dysbiosis are highly dynamic and influenced by multiple factors, including radiation dose, dose rate, fractionation schedule, exposure site, sampling time, host genetics, and underlying disease status (Lu et al., 2025; Li et al., 2022; Iacovacci et al., 2024). Experimental studies generally indicate that microbiota alterations occur in a dose- and time-dependent manner. Following total-body irradiation, changes in community composition frequently precede measurable reductions in alpha diversity, indicating that taxonomic remodeling occurs before substantial loss of microbial richness (Lu et al., 2025; Zhang et al., 2023). During the peak phase of intestinal injury, microbial diversity declines significantly, accompanied by reduced community stability and enrichment of stress-tolerant microorganisms. Partial recovery may occur during later stages; however, persistent alterations in specific taxa and microbial functions are commonly observed (Guo et al., 2020; Lu et al., 2025). Accumulating evidence further indicates that radiation delivery patterns substantially influence the severity of dysbiosis. Compared with fractionated irradiation, acute single-dose exposure generally induces more pronounced disruption of microbial diversity, greater depletion of beneficial microbial metabolites, impaired epithelial barrier function, and increased intestinal permeability (Wang et al., 2024; Lu et al., 2025, 2024). These findings suggest that fractionated radiation permits partial recovery of both the intestinal epithelium and microbial ecosystem between treatment cycles, thereby mitigating microbiota disruption despite comparable cumulative doses.

Several clinical observations partially parallel findings from experimental models. Patients receiving abdominal or pelvic radiotherapy consistently exhibit reduced microbial diversity and significant shifts in microbial community composition, particularly those who develop radiation-induced diarrhea or enteritis (Iacovacci et al., 2024; Ma et al., 2025; Khorashadizadeh et al., 2024). Greater microbial instability has been associated with increased gastrointestinal toxicity, suggesting that baseline microbial composition and ecosystem resilience may influence individual susceptibility to radiation injury (Iacovacci et al., 2024; Ma et al., 2025). Moreover, systematic reviews have identified that reduced microbial diversity is one of the most consistent microbial features associated with radiation-induced gastrointestinal complications (Lu et al., 2024; Yang et al., 2023; Khorashadizadeh et al., 2024; Wang et al., 2025). Collectively, these findings demonstrate that alterations in microbial diversity may reflect tissue injury while also identifying ecological states associated with toxicity. Changes in microbial diversity and community stability therefore represent promising biomarkers for predicting radiation-induced gastrointestinal toxicity and provide a biological foundation for microbiota-targeted interventions aimed at preserving intestinal homeostasis and improving radiation tolerance.

### 3.2 Radiation-associated taxonomic alterations

Radiation-induced dysbiosis is characterized by a reproducible pattern of taxonomic remodeling, involving the depletion of beneficial commensal bacteria and the expansion of opportunistic pathobionts (Crawford and Gordon, 2005; Li et al., 2020). Although the magnitude of these alterations varies across radiation models, doses, host backgrounds, and clinical settings, this ecological shift has been consistently observed in both experimental and clinical studies (Lu et al., 2025; Zhang et al., 2023; Iacovacci et al., 2024).

#### 3.2.1 Depletion of beneficial commensals

Irradiation frequently reduces the abundance of beneficial commensal bacteria involved in maintaining intestinal homeostasis, including members of the families Lachnospiraceae, Ruminococcaceae, and Muribaculaceae, as well as the genera Faecalibacterium, Bifidobacterium, Lactobacillus, and Akkermansia (Guo et al., 2020; Li et al., 2020; Lu et al., 2025; Li et al., 2022). These microorganisms contribute to carbohydrate fermentation, production of microbiota-derived metabolites, maintenance of epithelial barrier integrity, and regulation of mucosal immune homeostasis (Guo et al., 2020; Li et al., 2020). Their depletion is therefore associated with impaired microbial metabolic capacity, reduced epithelial resilience, weakened barrier function, and diminished resistance to inflammation. Among these taxa, Lachnospiraceae, Ruminococcaceae, and Faecalibacterium represent major producers of short-chain fatty acids (SCFAs) (Louis and Flint, 2017; Koh et al., 2016), whereas Bifidobacterium and Lactobacillus play important roles in maintaining microbial balance and mucosal defense. Muribaculaceae, a dominant murine commensal family, is similarly associated with carbohydrate metabolism and microbial homeostasis (Guo et al., 2020; Lu et al., 2025). In contrast, the role of Akkermansia muciniphila appears more context-dependent (Yu et al., 2025; He et al., 2023). Although it generally supports mucus turnover and barrier maintenance, excessive expansion under conditions of severe mucosal injury may further accelerate mucus degradation. Collectively, depletion of these beneficial microorganisms represents one of the most consistent microbial signatures of radiation-induced dysbiosis (Guo et al., 2020; Li et al., 2020; Lu et al., 2025; Zhang et al., 2023).

#### 3.2.2 Enrichment of opportunistic pathobionts

Concomitant with the loss of beneficial anaerobes, irradiation frequently promotes the expansion of opportunistic and pro-inflammatory bacteria, most notably members of the phylum Proteobacteria and genera such as Escherichia-Shigella, Enterococcus, and Desulfovibrio (Li et al., 2020; Lu et al., 2025; Zhang et al., 2023). The enrichment of Proteobacteria is widely regarded as a hallmark of microbial ecosystem instability and inflammatory dysbiosis (Li et al., 2020; Lu et al., 2024), reflecting the altered intestinal microenvironment following radiation exposure. Many of these pathobionts are associated with increased endotoxin production, disruption of epithelial barrier integrity, and amplification of mucosal inflammation, thereby contributing to sustained intestinal injury. However, not all radiation-associated bacterial changes can be simply classified as beneficial or detrimental. For example, the role of Enterococcus remains controversial, with different strains exhibiting distinct metabolic and immunological properties (Iacovacci et al., 2024; Khorashadizadeh et al., 2024). Depending on the host immune status, radiation conditions, antibiotic exposure, and microbial community context, Enterococcus may either contribute to epithelial injury or participate in host adaptation and recovery. These observations underscore the importance of moving beyond genus-level classification toward strain-resolved and function-oriented analyses to better define the biological significance of radiation-associated microbial alterations.

### 3.3 Factors influencing microbiota responses to radiation

The response of the gut microbiota to irradiation is highly heterogeneous and is shaped by the combined effects of radiation-related, host-related, temporal, and environmental factors (Li et al., 2022; Lu et al., 2024). These variables influence both the magnitude and persistence of microbial dysbiosis, contributing to the considerable variability observed across experimental models and clinical studies.

#### 3.3.1 Radiation-related factors

Radiation dose, dose rate, fractionation schedule, and exposure site are major determinants of microbiota disruption (Lu et al., 2025, 2024). In general, higher radiation doses induce more severe and persistent dysbiosis, whereas lower doses are more likely to cause transient alterations. Fractionated irradiation generally results in less pronounced microbial disruption than single-dose exposure, likely because intermittent treatment permits partial recovery of the intestinal epithelium and microbial ecosystem between radiation sessions (Lu et al., 2025; Li et al., 2022). The anatomical site of irradiation further influences microbial responses. Abdominal and pelvic irradiation directly perturbs the intestinal microenvironment, whereas total-body irradiation produces additional systemic effects through simultaneous injury to multiple organs. Even non-abdominal irradiation may indirectly alter the gut microbiota through systemic inflammatory and neuroendocrine responses (Luo et al., 2022; Lu et al., 2024), highlighting the complex interaction between local and systemic radiation effects.

#### 3.3.2 Temporal dynamics

Radiation-induced microbial dysbiosis is a dynamic process rather than a static event. Early after irradiation, alterations are often characterized by compositional shifts with relatively preserved microbial richness. During the peak phase of tissue injury, microbial diversity declines markedly and community instability becomes most evident (Lu et al., 2025; Zhang et al., 2023). Although partial restoration of microbial composition may occur during recovery, persistent alterations in specific taxa and microbial functions are frequently observed. Consequently, longitudinal sampling is essential for distinguishing transient microbial perturbations from long-term ecological remodeling and for identifying microbial signatures associated with tissue recovery or chronic radiation injury.

#### 3.3.3 Host and environmental factors

Host characteristics further contribute to the heterogeneity of microbiota responses. Experimental variables such as genetic background, age, sex, diet, housing conditions, and baseline microbial composition can substantially influence radiation-induced dysbiosis in animal models (Li et al., 2022; Iacovacci et al., 2024). In clinical settings, treatment-related factors—including cancer type, radiotherapy regimen, concurrent chemotherapy, nutritional status, and concomitant medications—also shape microbiota alterations (Iacovacci et al., 2024; Ma et al., 2025; Yu et al., 2025; Khorashadizadeh et al., 2024). Among these variables, antibiotic exposure represents one of the most important confounding factors because of its profound and prolonged effects on microbial composition and function (Lu et al., 2024; Khorashadizadeh et al., 2024). Therefore, future studies should emphasize standardized experimental protocols, careful control of dietary and medication variables, detailed reporting of radiation parameters, and baseline microbiota profiling to improve reproducibility and facilitate cross-study comparisons.

### 3.4 Bidirectional interactions between radiation and the gut microbiota

The interaction between ionizing radiation and the gut microbiota is fundamentally bidirectional (Wang et al., 2024; Crawford and Gordon, 2005; Lu et al., 2024). Radiation disrupts the intestinal microenvironment, leading to microbial dysbiosis, while the altered microbiota subsequently shapes host susceptibility to radiation injury, tissue repair, and systemic recovery. This reciprocal relationship positions the gut microbiota as both a target and a regulator of radiation responses.

#### 3.4.1 Radiation-driven alterations of the intestinal microenvironment

Radiation profoundly remodels the ecological niche in which intestinal microorganisms reside. Loss of proliferating epithelial cells and goblet cells reduces mucus production and alters the availability of host-derived glycans, whereas Paneth-cell dysfunction can modify antimicrobial peptide secretion. Barrier disruption and inflammatory cell recruitment change local cytokine and antimicrobial pressure. At the same time, epithelial mitochondrial dysfunction and reduced epithelial oxygen consumption may increase oxygen availability near the mucosal surface, favoring facultative anaerobes over obligate anaerobic commensals. Radiation-associated malabsorption, epithelial debris, altered intestinal motility, and changes in bile acid metabolism further reshape nutrient availability and microbial selection (Hauer-Jensen et al., 2014; Moraitis et al., 2023; Wang et al., 2024).

These ecological changes have functional consequences for host–microbe metabolic crosstalk. Loss of fiber-fermenting anaerobes can decrease SCFA production, whereas disruption of microbial tryptophan and bile acid metabolism alters aryl hydrocarbon receptor (AhR)-, farnesoid X receptor (FXR)-, and Takeda G protein-coupled receptor 5 (TGR5)-dependent host signaling. Reduced microbial metabolites can subsequently impair epithelial energy metabolism, mucus production, immune tolerance, and regenerative responses. Thus, radiation-induced dysbiosis is best understood as the ecological consequence of a profoundly altered intestinal habitat that can subsequently feed back to amplify host injury (Koh et al., 2016; Zelante et al., 2013; Jiao et al., 2025; Cheng et al., 2026).

#### 3.4.2 Microbiota-mediated regulation of host radiation responses

In turn, the gut microbiota may influence the severity and outcome of radiation injury (Wang et al., 2024; Crawford and Gordon, 2005; Guo et al., 2020). Microbial dysbiosis has been associated with impaired epithelial barrier function, enhanced inflammatory responses, and delayed tissue regeneration, whereas selected microbial communities and microbiota-targeted interventions have been linked to improved intestinal homeostasis and radiation tolerance (Guo et al., 2020; Lu et al., 2024; Wang et al., 2025). These effects may involve microbiota-derived metabolites that influence epithelial repair, immune regulation, antioxidant defense, and metabolic homeostasis (Guo et al., 2020; Koh et al., 2016; Parada Venegas et al., 2019). However, radiation-associated taxonomic shifts should initially be interpreted as ecological signatures rather than causal determinants of radiation outcomes. Microbial taxa, community configurations, or metabolic features should be designated as functional modifiers only when their effects have been directly evaluated through microbiota transfer, microbial depletion followed by reconstitution, defined microbial colonization, metabolite manipulation, or pathway-dependent rescue experiments.

#### 3.4.3 Systemic and inter-organ communication

The biological consequences of radiation–microbiota interactions extend beyond the gastrointestinal tract. Radiation-induced dysbiosis and alterations in microbial metabolites can influence distant organs through gut–organ communication networks, including the gut–bone marrow, gut–lung, and gut–brain axes (Luo et al., 2022; Lu et al., 2024; Yang et al., 2023). These inter-organ interactions contribute to hematopoietic recovery, pulmonary inflammation, neuroimmune regulation, and other systemic responses following irradiation. Conversely, systemic radiation injury can further reshape the intestinal ecosystem, reinforcing the reciprocal relationship between the host and the gut microbiota (Yang et al., 2023; Wang et al., 2025). Collectively, radiation and the gut microbiota form a dynamic feedback network in which alterations in the intestinal microenvironment are associated with microbial dysbiosis, while experimental evidence indicates that selected microbial communities and metabolites can, in turn, modify host responses to radiation. This bidirectional framework provides the conceptual basis for investigating microbiota-associated radioprotection but does not imply that all reported microbial changes are causally involved in radiation outcomes. Importantly, the evidence supporting individual microbiota–host relationships varies substantially across experimental systems. In this review, we therefore distinguish five broad evidence categories: (I) human interventional evidence; (II) human observational evidence; (III) causally supported preclinical evidence based on direct microbiota or metabolite manipulation and/or pathway-level intervention; (IV) associative preclinical evidence derived predominantly from microbiome, metabolomic, or multi-omics profiling; and (V) mechanistic hypotheses extrapolated from non-radiation biological settings. Terms implying functional mediation are reserved for relationships supported by direct experimental manipulation, whereas findings derived primarily from compositional or multi-omics associations are described as radiation-associated signatures, candidate mediators, or proposed mechanisms. Representative examples across these evidence categories, together with their causal-validation approaches and current translational status, are summarized in Table 1.

**Table 1:** Evidence hierarchy, causal-validation approaches, and translational maturity of representative microbiota-associated factors and interventions in radiation-induced injury.

Factor or strategy	Representative evidence	Main experimental system	Causal-validation approach	Evidence category	Current translational status
Radiation-associated microbial taxa	16S rRNA sequencing, metagenomic and multi-omics signatures associated with radiation exposure or toxicity	Human observational cohorts and animal profiling studies	Generally absent; predominantly compositional or multi-omics association	II or IV, depending on the study system	Biomarker discovery
Selected SCFAs, particularly propionate and butyrate	Radiation-survivor multi-omics studies combined with direct metabolite supplementation	Mainly irradiated mice	Direct metabolite administration; pathway necessity not uniformly established	III for intervention studies; IV for profiling-derived associations	Preclinical
*Faecalibaculum rodentium*–butyrate relationship	Bacterial administration and butyrate supplementation following irradiation	Mice	Direct bacterial and metabolite intervention with assessment of barrier and metabolic outcomes	III	Preclinical
*Akkermansia muciniphila*−3HB–GPR43/IL-6 relationship	Patient-associated metabolic observations combined with microbial, metabolite, cellular and animal experiments	Human observational samples, cells and mice	*A. muciniphila* administration, 3HB supplementation and pathway-level evaluation	II for human association + III for preclinical functional validation	Preclinical mechanism supported by human association
I3A–AhR/IL-10/Wnt relationship	Irradiated intestinal organoids and animal models	Organoids and irradiated animals	I3A administration with pathway-level intervention or validation	III	Preclinical
LCA-associated ferroptosis regulation	Cellular, animal and human-organoid experiments	Cells, mice and human organoids	LCA intervention with ferroptosis- and lipid-remodeling-related pathway validation	III	Preclinical
Broader microbiota–lipid metabolism–ferroptosis axis	Multi-omics associations and selected mechanistic studies	Mainly animal and cellular models	Incomplete; direct validation is restricted to selected metabolite–ferroptosis pathways	III for selected branches; IV–V for the broader proposed axis	Exploratory mechanistic framework
Probiotics	Randomized trials, meta-analyses and strain-specific animal studies	Humans and animals	Human efficacy testing for selected formulations; mechanisms remain strain-specific	I for selected formulations + III for strain-specific mechanisms	Comparatively greater clinical support, but not standardized therapy
Dietary and prebiotic interventions	Fiber, inulin and phytate intervention studies	Mainly animals	Direct dietary intervention; microbiota dependence variably tested	III	Predominantly preclinical
FMT/WMT	Early human studies and community-transfer experiments	Humans and animals	Community transfer supports microbiota dependence; human efficacy remains unconfirmed	Limited I/II + III	Investigational
Postbiotics and defined microbial metabolites	Direct supplementation of selected metabolites	Mainly animals, cells and organoids	Direct metabolite intervention; receptor or pathway necessity variably tested	III for selected metabolites	Preclinical
Engineered probiotics	Propionate-producing *Escherichia coli* Nissle 1917	Mice	Direct administration of a programmable microbial therapeutic	III	Preclinical proof-of-concept
Microbiota-oriented nanomedicine	Delivery-platform and microbiota-modulating formulation studies	Experimental animal and cellular models	Variable; microbiota dependence is often untested	III only when dependence is tested; otherwise IV–V	Exploratory

Evidence categories were defined as follows: I, human interventional evidence; II, human observational evidence; III, causally supported preclinical evidence based on direct microbial or metabolite manipulation and/or pathway-level intervention; IV, associative preclinical evidence derived primarily from microbiome, metabolomic, or multi-omics profiling; and V, mechanistic hypotheses extrapolated from non-radiation biological contexts. These categories describe the type and relative strength of evidence and should not be interpreted as a formal GRADE assessment. Evidence strength may differ among individual studies within the same intervention class.

3HB, 3-hydroxybutyrate; AhR, aryl hydrocarbon receptor; FMT, fecal microbiota transplantation; GPR43, G protein-coupled receptor 43; I3A, indole-3-acetic acid; IL-6, interleukin-6; LCA, lithocholic acid; SCFAs, short-chain fatty acids; WMT, washed microbiota transplantation.

#### 3.4.4 Methodological heterogeneity and limitations of current evidence

Despite broadly convergent evidence that irradiation perturbs the gut microbiota, substantial heterogeneity exists among preclinical studies. Experimental protocols differ markedly in radiation source and quality, total dose, dose rate, single vs. fractionated exposure, total-body vs. localized irradiation, intestinal segment examined, sampling time, mouse strain, sex, age, diet, housing conditions, and baseline microbiota. These variables can independently influence both radiation sensitivity and microbial ecology, thereby limiting direct comparisons across studies. In particular, commonly used high-dose total-body or abdominal irradiation models reproduce acute gastrointestinal or hematopoietic radiation syndromes but do not fully recapitulate the spatially restricted, fractionated exposure typically encountered during clinical radiotherapy. Consequently, microbial signatures identified in one experimental setting should not automatically be considered universal biomarkers of radiation injury (Lu et al., 2025; Palkovsky et al., 2025; Li et al., 2022).

Translation is further complicated by fundamental differences between murine and human microbiomes, host metabolism, immune physiology, dietary exposure, and treatment context. Most animal experiments are performed in genetically homogeneous animals under controlled diets and environments, whereas patients receiving radiotherapy constitute heterogeneous populations with different malignancies, radiation fields, concurrent chemotherapy or immunotherapy, medications, nutritional status, and baseline microbiota. Human evidence remains dominated by relatively small observational cohorts, and only a limited number of microbiota-directed interventions have undergone randomized evaluation. Accordingly, associations observed in clinical cohorts cannot establish whether microbial changes contribute to toxicity, reflect tissue injury, or arise from treatment-related confounding factors. Future studies would benefit from harmonized reporting of irradiation parameters, longitudinal sampling, baseline microbiome characterization, standardized toxicity endpoints, multi-center validation, and adequately powered randomized controlled trials (Iacovacci et al., 2024; Ma et al., 2025; Palkovsky et al., 2025; Khorashadizadeh et al., 2024).

## 4 Microbiota-derived metabolites involved in radiation-induced injury

Microbiota-derived metabolites constitute an important molecular interface between the intestinal microbiota and host physiology and have emerged as candidate regulators of radiation responses. Radiation-associated dysbiosis is accompanied by alterations in several metabolic classes, including short-chain fatty acids (SCFAs), tryptophan-derived metabolites, bile acids, lipid-related metabolites, polyamines, and other bioactive molecules. However, the strength of evidence supporting their involvement in radiation injury is highly heterogeneous (Guo et al., 2020; Li et al., 2020; Koh et al., 2016; Zelante et al., 2013; Jiao et al., 2025; Mafe and Büsselberg, 2026).

Interpretation of radiation-associated metabolomic studies nevertheless requires several methodological considerations. First, metabolite abundance differs substantially between mice and humans because of species-specific microbiota composition, diet, bile acid pools, hepatic metabolism, intestinal transit, and host–microbial co-metabolism. Therefore, metabolite signatures identified in murine irradiation models cannot be assumed to quantitatively reproduce those observed in patients. Second, untargeted metabolomics is influenced by sample type, collection and storage procedures, extraction methods, chromatographic platform, ionization mode, spectral libraries, batch effects, and metabolite annotation confidence. A substantial proportion of detected features remain incompletely annotated, whereas relative ion abundance does not necessarily reflect absolute metabolite concentration or biological flux (Sumner et al., 2007).

**Figure 2:**
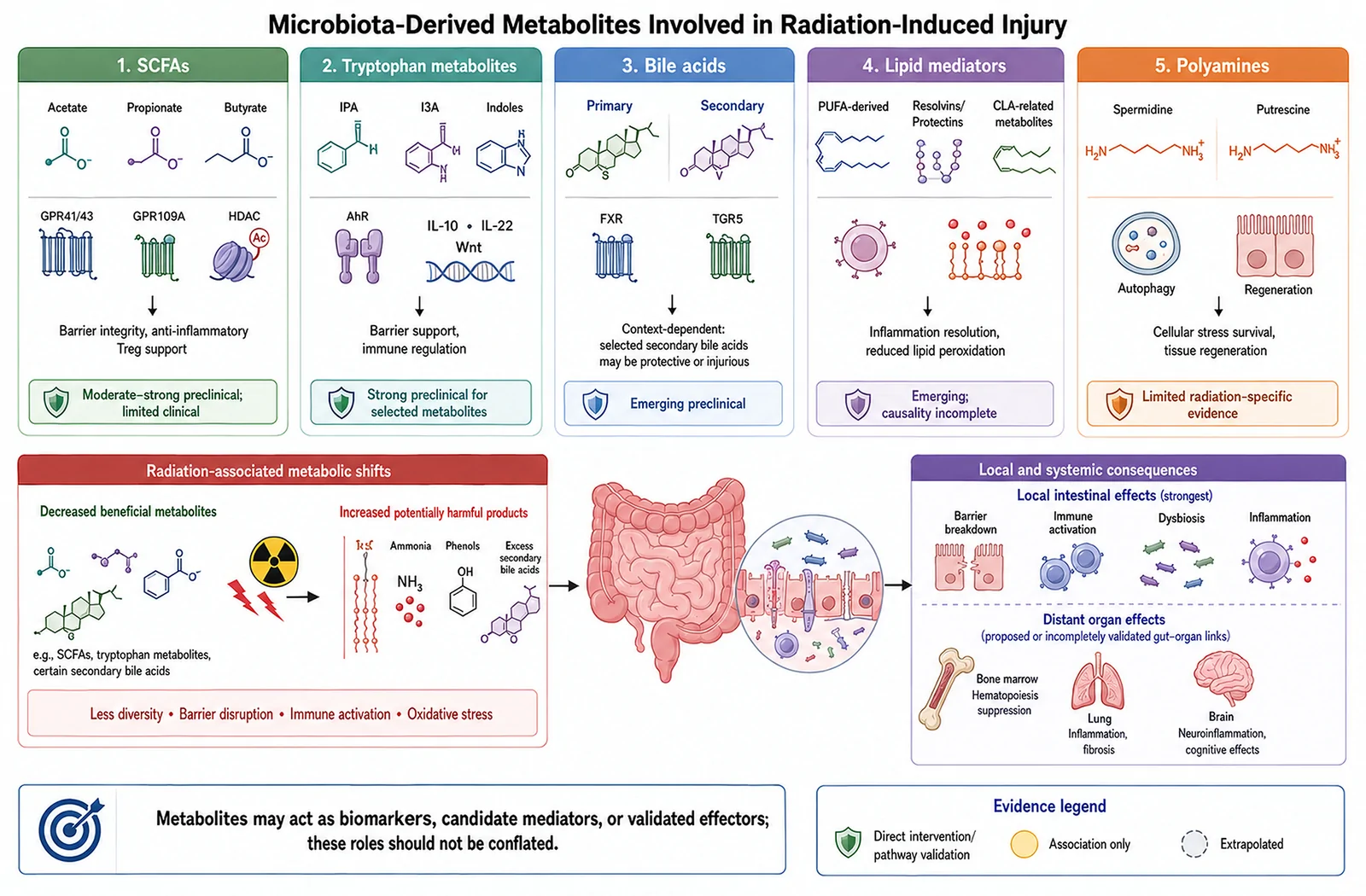
Evidence-aware overview of microbiota-associated metabolites in radiation-induced injury. Irradiation and radiation-associated intestinal dysbiosis are accompanied by alterations in short-chain fatty acids, tryptophan-derived metabolites, bile acids, microbiota-associated lipid mediators, polyamines, and potentially harmful microbial products. These metabolite classes may be associated with epithelial barrier integrity, immune regulation, oxidative stress, lipid peroxidation, and tissue repair; however, the strength and nature of radiation-specific evidence vary substantially among individual metabolites and pathways. Selected metabolites have been functionally evaluated through direct supplementation, microbial manipulation, and/or pathway-level intervention, whereas many other reported changes remain associative metabolic signatures or candidate mediators identified by metabolomic profiling. Local intestinal effects are generally supported more consistently than distant gut–organ effects, for which causal validation remains limited. Bile acids and lipid mediators are context dependent and may exert protective or injurious effects according to molecular species, concentration, tissue, timing, and radiation setting. Evidence symbols distinguish direct intervention and/or pathway-level validation, association-only evidence, and mechanisms extrapolated from non-radiation biological contexts. Abbreviations: AhR, aryl hydrocarbon receptor; CLA, conjugated linoleic acid; FXR, farnesoid X receptor; GPR, G protein-coupled receptor; HDAC, histone deacetylase; I3A, indole-3-acetic acid; IPA, indole-3-propionic acid; PUFA, polyunsaturated fatty acid; SCFAs, short-chain fatty acids; TGR5, Takeda G protein-coupled receptor 5.

Most importantly, an altered metabolite may represent a biomarker of radiation-induced ecological or tissue damage rather than a causal mediator. Stronger causal inference requires evidence that manipulation of the metabolite alters radiation outcome and, ideally, that the relevant host receptor or signaling pathway is necessary for this effect. Stable-isotope tracing, targeted quantitative metabolomics, microbial genetic manipulation, receptor-deficient models, and rescue experiments will therefore be important for distinguishing metabolic biomarkers from mechanistically active mediators. Figure 2 summarizes the major microbiota-associated metabolite classes while distinguishing selected functionally tested effectors from associative biomarkers and candidates extrapolated from related biological contexts (Blake et al., 2024; Wang et al., 2024).

### 4.1 Short-chain fatty acids

SCFAs, primarily acetate, propionate, and butyrate, are the major fermentation products of dietary fiber generated by anaerobic gut bacteria (Louis and Flint, 2017; Koh et al., 2016). They are predominantly produced by members of the Lachnospiraceae and Ruminococcaceae families, together with genera such as Faecalibacterium, Roseburia, Eubacterium, Bifidobacterium, and Lactobacillus (Louis and Flint, 2017). Radiation-induced depletion of SCFA-producing taxa has frequently been accompanied by reduced intestinal SCFA levels in experimental models. (Guo et al., 2020; Li et al., 2020; Lu et al., 2025). Among SCFAs, butyrate serves as the primary energy source for colonocytes and is essential for maintaining epithelial homeostasis, whereas acetate and propionate contribute predominantly to immune and metabolic regulation (Koh et al., 2016; Tan et al., 2014). Collectively, SCFAs preserve intestinal barrier integrity, support epithelial regeneration, regulate mucosal immune homeostasis, and limit excessive inflammatory responses, thereby maintaining intestinal resilience following radiation exposure (Koh et al., 2016; Parada Venegas et al., 2019).

Multi-omics and metabolomic studies have repeatedly identified alterations in SCFA-related metabolic profiles after irradiation, but such associations alone do not establish that endogenous SCFA depletion causes radiation injury. A representative example is the study by Guo et al., in which radiation-surviving mice exhibited enrichment of specific bacterial taxa together with increased fecal propionate and tryptophan-related metabolites. Importantly, this study progressed beyond association by demonstrating that supplementation with selected metabolites improved gastrointestinal and hematopoietic recovery in irradiated mice (Guo et al., 2020). Thus, propionate represents a candidate mediator supported by both associative multi-omics evidence and functional preclinical intervention.

More recent studies have provided additional causal support for selected SCFA-producing microorganisms. Supplementation with Faecalibaculum rodentium or its metabolite butyrate attenuated radiation-induced intestinal barrier dysfunction and supported hematopoietic recovery in mice, providing direct preclinical evidence for a F. rodentium–butyrate axis (Zhu et al., 2026). Similarly, an engineered Escherichia coli Nissle 1917 strain capable of sustained propionate production ameliorated abdominal radiation-induced intestinal injury in mice, although this approach remains entirely preclinical (Gao et al., 2026). Collectively, these studies place SCFAs among the comparatively better-supported preclinical candidate mediators of microbiota-associated radioprotection. Nevertheless, evidence that endogenous SCFA production is necessary for radiation resistance remains incomplete, and controlled clinical studies directly testing SCFA-based radioprotection in patients are currently lacking.

### 4.2 Tryptophan-derived metabolites

Microbial tryptophan metabolism represents another major pathway through which the gut microbiota regulates host responses to radiation (Zelante et al., 2013; Xie et al., 2024). In addition to host metabolism through the kynurenine and serotonin pathways, gut microorganisms convert dietary tryptophan into a variety of bioactive indole derivatives, including indole-3-acetic acid (IAA), indole-3-propionic acid (IPA), indole-3-aldehyde (IAld), and indole-3-lactic acid (ILA) (Zelante et al., 2013; Rosser et al., 2020). These metabolites exert both local and systemic effects and are increasingly recognized as candidate mediators of microbiota-associated radiation responses (Xie et al., 2024; Wang et al., 2025).

Many biological effects of microbial indole metabolites can involve aryl hydrocarbon receptor (AhR) signaling; however, the extent of radiation-specific validation differs among individual metabolites. Through AhR-dependent signaling, microbial indole metabolites promote intestinal barrier maintenance, support epithelial repair, enhance immune homeostasis, and facilitate recovery following radiation exposure (Zelante et al., 2013; Xie et al., 2024; Jia et al., 2025). In addition, several tryptophan-derived metabolites possess intrinsic antioxidant and anti-inflammatory properties, further contributing to protection against radiation-induced oxidative stress and tissue injury (Xie et al., 2024; Wang et al., 2025).

The biological significance of radiation-associated tryptophan metabolism should be interpreted according to the underlying experimental evidence. Multi-omics studies have identified increased tryptophan-related metabolites in radiation-surviving animals, providing association-based evidence that this metabolic pathway may accompany radiation resilience (Guo et al., 2020). Functional validation is stronger for selected metabolites. For example, microbiota-derived indole-3-carboxaldehyde (I3A) protected intestinal organoids and irradiated mice, and pathway-intervention experiments implicated AhR/interleukin-10 (IL-10)/Wnt signaling in this effect (Xie et al., 2024). Similarly, indole-3-propionic acid has been functionally linked to protection against radiation toxicity in experimental models.

These findings support a functional role for selected indole metabolites in preclinical radiation injury, but they should not be generalized to the entire tryptophan metabolite pool. Moreover, mechanisms such as AhR-dependent immune regulation and IL-22-mediated epithelial repair are well established in intestinal biology but have not been equivalently validated for every microbial indole metabolite under irradiation. Thus, microbial tryptophan metabolites should currently be regarded as mechanistically supported preclinical candidates, with the strength of causal evidence varying substantially among individual metabolites. Human interventional evidence remains absent, and future studies using defined metabolite-producing strains, microbiota depletion/reconstitution, and receptor-specific genetic models will be important for establishing necessity as well as sufficiency (Zelante et al., 2013; Xie et al., 2024; Lindemans et al., 2015; Kang et al., 2025).

### 4.3 Bile acids

Bile acids are increasingly recognized as important mediators of microbiota–host communication during radiation injury (Jiao et al., 2025; Cheng et al., 2026). Primary bile acids synthesized in the liver are converted by gut microbial enzymes into secondary bile acids, generating a diverse bile acid pool that regulates intestinal homeostasis, metabolism, immune function, and epithelial integrity (Cheng et al., 2026). Radiation-induced dysbiosis disrupts bile acid metabolism by altering microbial bile acid transformation and enterohepatic circulation, leading to changes in both intestinal and circulating bile acid profiles (Jiao et al., 2025). Conversely, bile acids influence microbial community composition through their antimicrobial activity, establishing a dynamic microbiota–bile acid feedback loop.

Bile acids signal through receptors including farnesoid X receptor (FXR) and Takeda G protein-coupled receptor 5 (TGR5) and regulate epithelial, metabolic, and immune functions. However, much of this broader receptor biology has been established outside radiation-specific models and should therefore not be interpreted as direct evidence of radioprotection. Radiation-specific evidence for the microbiota–bile acid axis is comparatively recent and remains predominantly preclinical. A notable advance is the demonstration that microbiota-associated lithocholic acid (LCA) can modulate radiation-induced intestinal ferroptosis. Recent work showed that LCA administration protected irradiated cells, mice, and human small-intestinal organoids and implicated TGR5-dependent lipid-metabolic remodeling in this effect (Ouyang et al., 2026). This provides relatively strong mechanistic evidence for a specific LCA-associated pathway, but it does not establish that the entire microbiota–bile acid axis is a general determinant of radiation tolerance. Other bile acids, including ursodeoxycholic acid (UDCA) and tauroursodeoxycholic acid (TUDCA), have biological properties relevant to tissue protection, yet their radiation-specific evidence is less extensive. Accordingly, the microbiota–bile acid axis should currently be considered an emerging, mechanistically supported preclinical target rather than an established clinical radioprotective pathway.

### 4.4 Lipid metabolites

Lipid metabolites are emerging as important regulators of radiation-induced injury and represent a growing area of interest in radiation microbiome research (Stockwell et al., 2017; Jiang et al., 2021; Doll et al., 2017; Lei et al., 2020). Ionizing radiation induces profound lipid metabolic remodeling through oxidative stress, mitochondrial dysfunction, membrane damage, and inflammatory activation (Zhao and Robbins, 2009; Mohammadgholi and Hosseinimehr, 2024). Multi-omics studies have identified covariation between radiation-associated dysbiosis and host lipid remodeling, supporting lipidomic signatures as candidate biomarkers but not establishing a causal microbiota–lipid relationship (Guo et al., 2020; Li et al., 2020). Radiation-induced lipid remodeling primarily involves changes in glycerophospholipids, sphingolipids, and polyunsaturated fatty acid metabolism (Kagan et al., 2017; Doll et al., 2017), all of which are essential for membrane integrity, cellular signaling, inflammatory regulation, and tissue repair. Dysregulation of these lipid pathways contributes to epithelial injury, vascular dysfunction, and persistent inflammation following irradiation. Among these processes, lipid peroxidation and ferroptosis have emerged as key mechanisms linking oxidative stress with radiation-induced tissue damage (Dixon et al., 2012; Stockwell et al., 2017; Jiang et al., 2021; Lei et al., 2020). Excessive accumulation of oxidized membrane lipids promotes ferroptotic cell death and has been implicated in intestinal, hematopoietic, and other radiation-sensitive tissues (Lei et al., 2020; Zhang et al., 2022; Cui et al., 2025).

Current evidence clearly supports lipid peroxidation and ferroptosis as components of radiation-induced tissue injury, but the evidence directly linking the gut microbiota to these processes is substantially less mature. Microbiome–lipidomic studies predominantly demonstrate covariation between microbial alterations and host lipid remodeling and therefore cannot determine whether dysbiosis drives ferroptosis, results from radiation-induced tissue injury, or participates in a bidirectional feedback loop. Selected metabolite-intervention studies now provide proof-of-concept that microbial metabolism can influence ferroptosis in radiation models. In particular, microbiota-associated LCA has been functionally linked to reduced radiation-induced ferroptotic injury (Ouyang et al., 2026). Nevertheless, comparable causal evidence is lacking for most radiation-associated taxa, glycerophospholipid species, and microbial lipid-related metabolites. We therefore regard the microbiota–lipid metabolism–ferroptosis axis as an emerging mechanistic framework with selected experimentally validated components, rather than a fully established causal pathway. Future studies combining microbiota depletion or gnotobiotic approaches with lipidomics and ferroptosis-specific genetic or pharmacological interventions will be required to resolve directionality and causality.

Increasing evidence indicates that the gut microbiota plays an important role in shaping host lipid metabolism (Guo et al., 2020; Li et al., 2020; Cui et al., 2025). Through regulation of bile acid transformation, fatty acid metabolism, and the production of bioactive microbial metabolites, the gut microbiota influences lipid homeostasis, inflammatory responses, and susceptibility to lipid peroxidation (Cui et al., 2025; Mao et al., 2024). Accordingly, disruption of microbiota–lipid interactions may contribute to enhanced radiosensitivity, whereas restoration of microbial metabolic function may attenuate lipid metabolic dysfunction and ferroptosis. Although research in this area is still emerging, recent studies have highlighted the therapeutic potential of targeting the microbiota–lipid metabolism axis (Lei et al., 2020; Chen et al., 2024; Li et al., 2025; Huang et al., 2024). Modulation of lipid metabolic pathways has shown promise in reducing ferroptosis, preserving epithelial integrity, and improving tissue repair following irradiation. These findings identify lipid metabolites as an emerging class of microbiota-regulated mediators and suggest that modulation of lipid metabolism may provide new opportunities for microbiota-based radioprotective strategies.

### 4.5 Polyamines and other microbial metabolites

In addition to SCFAs, tryptophan metabolites, bile acids, and lipid metabolites, several emerging microbiota-derived molecules have also been implicated in radiation responses, although their biological functions remain less well characterized. Among these, polyamines, including putrescine, spermidine, and spermine, represent the most extensively studied group (Mafe and Büsselberg, 2026). Produced by both host cells and gut microorganisms, polyamines are essential for cell proliferation, epithelial renewal, and tissue repair (Mafe and Büsselberg, 2026). Increasing evidence suggests that spermidine and related polyamines promote intestinal regeneration, reduce oxidative stress, and improve cellular resilience following irradiation. Accordingly, strategies aimed at increasing polyamine production through microbial modulation or metabolite supplementation may facilitate recovery from radiation-induced intestinal injury. Purine metabolites, particularly inosine, have recently emerged as important immunometabolic mediators (Guo et al., 2020; Zhu et al., 2025). Although direct evidence in radiation injury remains limited, inosine has been associated with enhanced immune function and is enriched in several radioprotective microbial communities, suggesting a potential role in systemic radiation tolerance (Guo et al., 2020).

A particularly informative example of evidence progression is provided by 3-hydroxybutyrate (3HB). Ge et al. first identified reduced 3HB levels in radiation proctopathy and reported an inverse association between 3HB and interleukin-6 (IL-6), including observations in patients receiving radiotherapy (Ge et al., 2024). Importantly, the study extended beyond clinical and metabolomic association: 3HB supplementation and Akkermansia muciniphila administration ameliorated radiation injury in experimental systems, while irradiated cell–fecal microbiota co-culture experiments and *in vivo* assays implicated G protein-coupled receptor 43 (GPR43)-mediated IL-6 signaling (Ge et al., 2024). Thus, this study provides an example in which human observational evidence was complemented by targeted preclinical functional validation. Nevertheless, the clinical observations themselves remain associative, and therapeutic efficacy of 3HB or A. muciniphila has not yet been established in controlled human intervention studies.

Other metabolites, including lactate (Lee et al., 2018), succinate (Wei et al., 2023), microbial outer membrane vesicles (OMVs), and microbially synthesized vitamins (Zhu et al., 2025), may also participate in radiation responses by regulating epithelial metabolism, immune homeostasis, and host–microbe communication. In addition, histidine-derived imidazole propionate has been implicated in protection against radiation-induced cardiopulmonary injury (Chen et al., 2021). However, current evidence for many of these metabolites remains largely indirect, and their specific contributions to radiation-induced injury require further investigation. Collectively, polyamines, purine metabolites, 3HB, lactate, succinate, microbial vesicles, and microbial vitamins should not be regarded as having equivalent evidence strength. Whereas selected metabolites such as 3HB have undergone targeted functional validation in radiation models, evidence for several others remains largely associative or extrapolated from non-radiation contexts. Their inclusion here therefore reflects emerging biological plausibility rather than established radioprotective efficacy.

## 5 Mechanistically supported and proposed pathways linking the gut microbiota to radiation responses

Rather than acting through isolated metabolite-specific pathways, microbiota-derived metabolites may converge on several host signaling hubs, including nuclear factor erythroid 2-related factor 2 (Nrf2), NF-κB/NLR family pyrin domain-containing 3 (NLRP3), AhR, FXR/TGR5, peroxisome proliferator-activated receptors (PPARs), and Wnt/β-catenin. Table 2 summarizes the representative metabolite classes, major host targets, biological outcomes, and relative strength of radiation-specific evidence associated with these convergent signaling networks. The following sections therefore focus on major biological outcomes while avoiding repetitive descriptions of overlapping signaling pathways (Koh et al., 2016; Zelante et al., 2013; Jiao et al., 2025; Cheng et al., 2026; Cui et al., 2025).

**Table 2:** Convergent host signaling pathways associated with microbiota-derived metabolites in radiation responses.

Metabolite class	Representative metabolites	Major host targets/pathways	Principal biological effects	Radiation-specific evidence
SCFAs	Acetate, propionate, butyrate	GPR41/43, GPR109A, HDACs; Nrf2 and NF-κB as context-dependent downstream pathways	Barrier maintenance, immune modulation, antioxidant responses, and epithelial recovery	Moderate-to-strong preclinical evidence for selected SCFAs; limited and heterogeneous clinical evidence
Tryptophan-derived metabolites	IPA, I3A, IAA, and other indole derivatives	AhR, IL-10, IL-22, Nrf2, and Wnt/β-catenin	Barrier support, epithelial regeneration, antioxidant responses, and mucosal immune regulation	Moderate-to-strong preclinical evidence for selected metabolites
Secondary bile acids	LCA, DCA, and other selected secondary bile acids	FXR, TGR5, and lipid-remodeling pathways	Context-dependent regulation of inflammation, metabolism, epithelial homeostasis, and ferroptosis; effects may be protective or injurious according to bile acid species, concentration, tissue, and timing	Emerging preclinical evidence; direct mechanistic support is restricted to selected bile acid–pathway relationships
Microbiota-associated lipid mediators	PUFA-derived mediators, CLA-related metabolites, and selected lipid-remodeling products	PPARs, Nrf2, and lipid-remodeling/ferroptosis-related pathways	Context-dependent modulation of inflammatory resolution, lipid homeostasis, oxidative stress, and lipid peroxidation	Emerging and predominantly preclinical; causal microbiota dependence remains incompletely established
Polyamines	Spermidine, putrescine	Autophagy-, antioxidant-, and regeneration-related pathways	Cellular stress adaptation, epithelial proliferation, and tissue repair	Limited radiation-specific evidence; much of the mechanistic rationale is extrapolated from non-radiation contexts
Multiple metabolite classes	Selected SCFAs, indole derivatives, bile acids, and other candidate metabolites	TLR4/NF-κB and NLRP3 inflammasome	Reduced inflammatory signaling and cytokine production in selected intervention models	Predominantly preclinical; pathway necessity and microbiota dependence are not uniformly established
Multiple metabolite classes	Selected SCFAs and tryptophan-derived metabolites, particularly I3A	AhR/IL-10/Wnt/β-catenin and related regenerative pathways	ISC-associated epithelial regeneration and mucosal repair in selected experimental systems	Moderate preclinical evidence for selected metabolite–pathway relationships; broader generalization remains unverified

The relative evidence descriptors used in this table represent a narrative assessment of radiation-specific evidence based on the experimental system, direct metabolite intervention, microbiota manipulation, pathway-level validation, and availability of human data. They do not constitute a formal GRADE assessment. Evidence strength may differ substantially among individual metabolites and pathways within the same metabolite class. Terms such as “moderate-to-strong,” “emerging,” and “limited” should therefore be interpreted comparatively rather than as standardized quantitative scores.

AhR, aryl hydrocarbon receptor; CLA, conjugated linoleic acid; DCA, deoxycholic acid; FXR, farnesoid X receptor; GPR, G protein-coupled receptor; HDACs, histone deacetylases; I3A, indole-3-acetic acid; IAA, indole-3-aldehyde; IL, interleukin; IPA, indole-3-propionic acid; ISC, intestinal stem cell; LCA, lithocholic acid; NF-κB, nuclear factor kappa B; NLRP3, NLR family pyrin domain-containing 3; Nrf2, nuclear factor erythroid 2-related factor 2; PPARs, peroxisome proliferator-activated receptors; PUFAs, polyunsaturated fatty acids; SCFAs, short-chain fatty acids; TGR5, Takeda G protein-coupled receptor 5; TLR4, Toll-like receptor 4.

**Figure 3:**
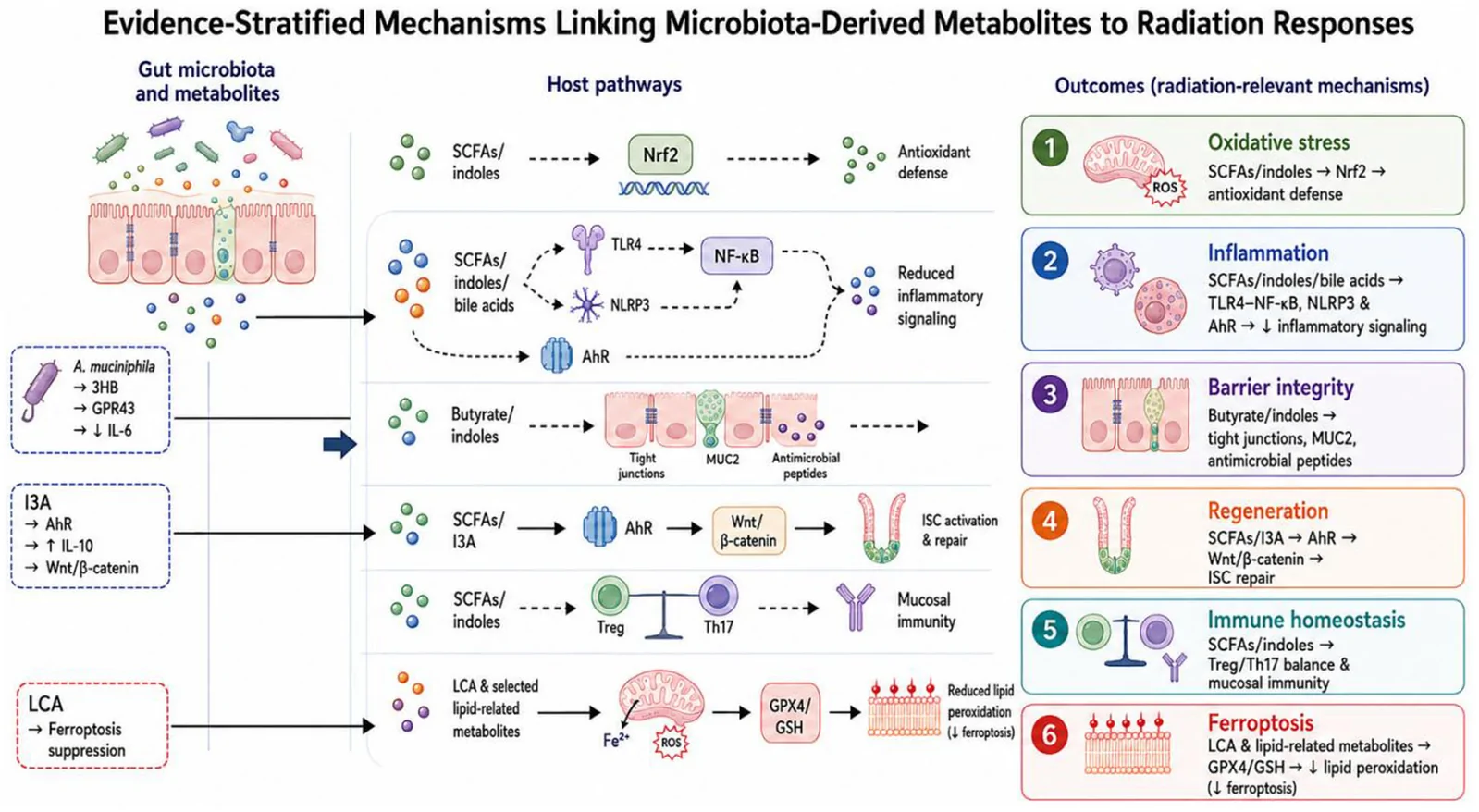
Evidence-stratified mechanisms linking microbiota-associated metabolites to radiation responses. Microbiota-associated metabolites may influence radiation-relevant biological outcomes through partially overlapping host pathways involving antioxidant defense, inflammatory signaling, epithelial barrier integrity, intestinal regeneration, immune homeostasis, and lipid peroxidation-associated ferroptosis. The strength of radiation-specific evidence differs across individual metabolite–pathway relationships. Solid arrows indicate direct mechanistic evidence from radiation models involving microbial or metabolite intervention together with pathway-level validation; dashed arrows indicate indirect or associative evidence obtained in radiation settings. The Akkermansia muciniphila−3-hydroxybutyrate–GPR43/IL-6 relationship and the I3A–AhR/IL-10/Wnt-associated regenerative pathway are shown as representative examples in which microbial or metabolite intervention has been combined with mechanistic evaluation. LCA-associated regulation of lipid peroxidation and ferroptosis is presented as a selected experimentally supported branch of the broader microbiota–lipid metabolism–ferroptosis framework. Other connections should be interpreted as candidate or context-dependent mechanisms rather than as uniformly established causal pathways. Abbreviations: 3HB, 3-hydroxybutyrate; AhR, aryl hydrocarbon receptor; GPR43, G protein-coupled receptor 43; GPX4, glutathione peroxidase 4; GSH, glutathione; I3A, indole-3-acetic acid; IL, interleukin; ISC, intestinal stem cell; LCA, lithocholic acid; NF-κB, nuclear factor kappa B; NLRP3, NLR family pyrin domain-containing 3; Nrf2, nuclear factor erythroid 2-related factor 2; ROS, reactive oxygen species; SCFAs, short-chain fatty acids; TLR4, Toll-like receptor 4; Treg, regulatory T cell.

Available evidence suggests that the gut microbiota and its metabolites may influence radiation responses through multiple, partially overlapping processes involving redox homeostasis, inflammation, epithelial barrier integrity, tissue regeneration, immune regulation, and lipid metabolism. However, the degree of radiation-specific mechanistic validation differs substantially among these pathways. Some relationships are supported by direct microbial or metabolite intervention and pathway perturbation, whereas others are inferred from radiation-associated multi-omics studies or from established host–microbiota biology in non-radiation settings. Accordingly, the following sections distinguish experimentally supported mechanisms from associative or proposed mechanistic links rather than presenting these pathways as equally established components of microbiota-mediated radioprotection. Whereas Table 2 provides a comparative overview across metabolite classes and convergent pathways, Figure 3 illustrates representative directional relationships between microbial metabolites, host signaling nodes, and radiation-relevant biological outcomes. The visual connections in Figure 3 should therefore not be interpreted as equivalent in causal strength (Blake et al., 2024; Wang et al., 2024; Guo et al., 2020; Xie et al., 2024; Ouyang et al., 2026).

### 5.1 Suppression of oxidative stress

Oxidative stress is one of the earliest and most critical events in radiation-induced injury (Zhao and Robbins, 2009; Mohammadgholi and Hosseinimehr, 2024). Excessive production of reactive oxygen species (ROS) following irradiation damages DNA, proteins, lipids, and mitochondria, leading to epithelial injury, inflammatory amplification, endothelial dysfunction, and impaired tissue regeneration (Zhao and Robbins, 2009; Mohammadgholi and Hosseinimehr, 2024; Rong et al., 2025). Therefore, limiting oxidative stress represents a central mechanism of radioprotection. Gut microbiota and their metabolites counteract radiation-induced oxidative injury through multiple complementary pathways. SCFAs, particularly butyrate and propionate, improve mitochondrial function and cellular energy metabolism, thereby reducing ROS accumulation and preserving epithelial homeostasis (Koh et al., 2016; Parada Venegas et al., 2019). Likewise, microbial tryptophan metabolites, including indole-3-propionic acid (IPA) and indole-3-acetic acid (IAA), exhibit antioxidant and cytoprotective activities that protect epithelial and hematopoietic tissues from oxidative damage (Zelante et al., 2013; Xie et al., 2024). Emerging evidence also suggests that bile acid signaling contributes to redox homeostasis by coordinating mitochondrial function, metabolism, and epithelial integrity (Jiao et al., 2025; Cheng et al., 2026).

Microbiota-associated metabolites have been linked to redox regulation after irradiation, although the strength of evidence varies. Functional studies support antioxidant and tissue-protective effects for selected metabolites, including propionate and specific tryptophan-derived compounds, whereas evidence for many broader microbiota–Nrf2 relationships remains indirect. For example, metabolite supplementation in irradiated animals has been associated with reduced oxidative damage and improved tissue recovery (Guo et al., 2020; Xie et al., 2024). These intervention studies support metabolite-dependent modulation of oxidative stress, but they do not establish Nrf2 as a universal downstream mediator of microbiota-based radioprotection. Nrf2 is a well-established endogenous antioxidant pathway in radiation biology and can also be influenced by microbial metabolites in other inflammatory and metabolic contexts. However, direct radiation-specific experiments demonstrating that microbiota-dependent protection requires Nrf2 are comparatively limited. Accordingly, Nrf2 should be regarded as a plausible and partially supported mechanistic node rather than a universally established microbiota-dependent pathway. Receptor- or Nrf2-deficient models combined with defined microbial or metabolite interventions will be required to establish pathway dependence.

### 5.2 Inhibition of inflammatory signaling

Excessive inflammation is a central driver of radiation-induced tissue injury (Zhao and Robbins, 2009; Mohammadgholi and Hosseinimehr, 2024). Following irradiation, epithelial damage, barrier disruption, and microbial translocation activate innate immune responses, leading to sustained production of pro-inflammatory cytokines and chemokines that amplify tissue damage and impair regeneration. Among the major inflammatory pathways involved, the TLR4/NF-κB axis and the NLRP3 inflammasome are considered key mediators of radiation-induced inflammatory responses. Microbiota-associated interventions can attenuate inflammatory responses after irradiation, but their upstream mechanisms are not uniformly defined. SCFA or indole-metabolite supplementation has been associated with reduced inflammatory cytokine production in experimental models, whereas probiotic and dietary interventions can simultaneously alter microbial composition, barrier integrity, and host immune signaling. Therefore, decreases in NF-κB or NLRP3 activation following these interventions should not automatically be interpreted as proof that microbiota-dependent protection is mediated exclusively through these pathways. More direct mechanistic evidence exists for selected microbe–metabolite–receptor relationships. For example, the A. muciniphila−3HB study combined clinical associations with experimental intervention and showed that the protective effect of 3HB involved GPR43-dependent regulation of IL-6 signaling (Ge et al., 2024). Such pathway-level validation provides stronger causal support than microbiome–cytokine correlations alone. By contrast, microbiota-dependent regulation of macrophage M1/M2 polarization and generalized TLR4/NF-κB/NLRP3 suppression remains supported largely by preclinical and, in some instances, non-radiation evidence. These pathways should therefore be interpreted as complementary candidate mechanisms whose relative contributions require direct testing in radiationmodels.

Across experimental radiation models, probiotic, dietary, natural-product, and metabolite interventions have frequently been accompanied by lower NF-κB/NLRP3 activation, reduced pro-inflammatory cytokine production, or shifts in macrophage phenotypes. These observations support inflammatory signaling as a recurrent treatment-responsive outcome, but they do not establish a common microbiota-dependent mechanism because many interventions also exert direct host effects. Macrophage polarization and inflammasome regulation should therefore be viewed as intervention-specific candidate mechanisms unless microbiota dependence and pathway necessity are demonstrated through microbial depletion, microbiota transfer, receptor-deficient models, or pathway-blocking experiments (Yang et al., 2023; Li et al., 2025; Devaraj et al., 2019; Ciorba et al., 2012; Riehl et al., 2019; Jian et al., 2022; Zhang et al., 2024; Li et al., 2010).

### 5.3 Restoration of intestinal barrier integrity

Disruption of the intestinal barrier is a hallmark of radiation-induced gastrointestinal injury and a major contributor to local inflammation and systemic toxicity (Hauer-Jensen et al., 2014; Wang et al., 2024). Irradiation impairs epithelial integrity, disrupts tight junctions, depletes mucus-producing goblet cells and antimicrobial peptide-secreting Paneth cells, and increases intestinal permeability. These changes facilitate bacterial translocation and perpetuate inflammation, creating a vicious cycle that delays mucosal repair.

Experimental intervention studies provide comparatively strong support for barrier restoration as a functional outcome of selected microbiota- and metabolite-based treatments. SCFA-producing bacteria, exogenous SCFAs, probiotics, and selected indole metabolites have been shown to preserve epithelial architecture or tight-junction-associated markers in irradiated animals (Guo et al., 2020; Zhu et al., 2026; Xie et al., 2024). However, improved barrier integrity is a biological outcome shared by multiple interventions and does not, by itself, demonstrate which microbial metabolite or receptor is causally required.

Evidence is particularly persuasive when microbial or metabolite manipulation is linked to a defined barrier phenotype. For example, F. rodentium and butyrate supplementation improved intestinal integrity after irradiation (Zhu et al., 2026). Conversely, proposed effects of microbial metabolites on Paneth-cell recovery, goblet-cell function, and antimicrobial peptide production are not equivalently validated across radiation models. We therefore interpret barrier restoration as a well-supported preclinical consequence of several microbiota-targeted interventions, while the specific molecular pathways remain intervention-dependent.

### 5.4 Promotion of epithelial regeneration and stem cell repair

Impaired epithelial regeneration is a defining feature of radiation-induced gastrointestinal injury. Because Lgr5^+^ intestinal stem cells (ISCs) sustain continuous epithelial renewal (Barker et al., 2007), their depletion following irradiation results in crypt loss, villus atrophy, and failure of mucosal repair (Barker et al., 2007; Qiu et al., 2010). Consequently, preservation and regeneration of the ISC compartment are central to recovery from radiation-induced intestinal injury. Gut microbiota and microbiota-derived metabolites facilitate epithelial regeneration by modulating key signaling pathways that govern ISC survival and tissue repair.

Wnt/β-catenin, epidermal growth factor receptor (EGFR), and cytokine-dependent pathways are established regulators of intestinal stem-cell biology, but direct evidence that the gut microbiota controls these pathways during radiation injury is uneven. The strongest conclusions should therefore be restricted to studies in which microbial metabolites were experimentally administered and regenerative pathways were functionally interrogated. For example, Xie et al. demonstrated that microbiota-derived I3A protected irradiated intestinal organoids and animals and implicated AhR/IL-10/Wnt signaling in this regenerative response (Xie et al., 2024). Such experiments provide direct preclinical support for an I3A-associated regenerative pathway. In contrast, broader statements that microbial metabolites generally activate Wnt, EGFR, or IL-22-dependent stem-cell regeneration extend beyond the available radiation-specific causal evidence. These pathways remain biologically plausible and are supported by intestinal regeneration literature, but their microbiota dependence after irradiation requires further validation using receptor-deficient, organoid, and defined microbial-metabolite systems (Lindemans et al., 2015; Wang et al., 2023; Kang et al., 2025).

### 5.5 Regulation of immune homeostasis

Radiation disrupts mucosal and systemic immune homeostasis, and microbiota-dependent modulation of this response is biologically plausible. However, direct radiation-specific evidence for several commonly proposed immune mechanisms remains less extensive than evidence from colitis, infection, and other inflammatory models. In particular, the ability of SCFAs to promote regulatory T-cell differentiation and influence regulatory T-cell (Treg)/T helper 17 (Th17) balance is well established in general mucosal immunology, but direct demonstration that this mechanism is necessary for SCFA-mediated radioprotection remains limited (Smith et al., 2013; Furusawa et al., 2013; Arpaia et al., 2013; Atarashi et al., 2013; Singh et al., 2014).

Similarly, microbial indole–AhR signaling can regulate epithelial and immune responses, but its contribution to radiation injury has been functionally validated only for selected metabolites and experimental contexts. We therefore distinguish established host–microbiota immunology from radiation-specific causal evidence. At present, immune homeostasis should be viewed as an important biological domain influenced by microbiota-targeted interventions, whereas the relative roles of Tregs, type 3 innate lymphoid cells (ILC3s), dendritic cells, macrophages, and secretory immunoglobulin A (IgA) in mediating radiation protection remain active areas of investigation (Zelante et al., 2013; Lindemans et al., 2015; Wang et al., 2023; Kang et al., 2025).

### 5.6 Modulation of ferroptosis and lipid peroxidation

Ferroptosis has emerged as an important mechanism underlying radiation-induced tissue injury (Dixon et al., 2012; Lei et al., 2020). This iron-dependent form of regulated cell death is driven by excessive lipid peroxidation and contributes to epithelial loss, mitochondrial dysfunction, inflammatory amplification, and impaired tissue regeneration following irradiation (Dixon et al., 2012; Yang et al., 2014; Stockwell et al., 2017; Jiang et al., 2021; Stockwell, 2022; Lei et al., 2020). Ferroptosis itself is increasingly supported as a component of radiation-induced intestinal injury, with radiation-specific studies implicating lipid peroxidation and regulators such as acyl-CoA synthetase long-chain family member 4 (ACSL4). However, this should be distinguished from the much less mature proposition that the gut microbiota broadly regulates radiation-induced ferroptosis.

Current evidence supports only selected links between microbial metabolism and ferroptotic responses. The recent demonstration that microbiota-associated LCA can suppress radiation-induced ferroptosis through TGR5-dependent lipid remodeling provides direct mechanistic support for one specific branch of this relationship (Ouyang et al., 2026). In contrast, proposed effects of SCFAs, tryptophan metabolites, microbial lipid remodeling, or dysbiosis-associated inflammatory signals on ferroptosis are supported to varying degrees by indirect radiation evidence or by studies from other disease contexts.

Accordingly, the microbiota–lipid metabolism–ferroptosis axis should presently be regarded as an emerging mechanistic framework rather than an established unified pathway. Its value lies in integrating several testable hypotheses concerning microbial metabolism, host lipid composition, oxidative stress, and ferroptotic susceptibility. Establishing causality will require experiments combining defined microbiota manipulation with ferroptosis-specific genetic or pharmacological perturbation (Cui et al., 2025; Lei et al., 2020; Ouyang et al., 2026).

## 6 Microbiota-based therapeutic opportunities: from clinical evidence to experimental platforms

The translational maturity of microbiota-based interventions for radiation-induced injury varies markedly. Probiotics have been evaluated in human interventional studies for selected radiotherapy-associated gastrointestinal toxicities, whereas FMT and washed microbiota transplantation (WMT) are supported only by limited early clinical investigations in radiation enteritis. In contrast, most defined microbial metabolites, prebiotic/dietary strategies, microbiota-active natural products, engineered probiotics, and microbiota-oriented nanomedicines remain predominantly preclinical in the radiation setting. These approaches should therefore not be interpreted as being at equivalent stages of clinical development. In the following sections, we distinguish clinical-supportive, investigational, preclinical, and exploratory strategies and consider efficacy together with safety, standardization, manufacturing, regulatory requirements, and potential interactions with anticancer treatment (Wang et al., 2024; Devaraj et al., 2019; Ding et al., 2020; Tao et al., 2026; Li et al., 2025; Wang et al., 2025; Zhang et al., 2025).

**Figure 4:**
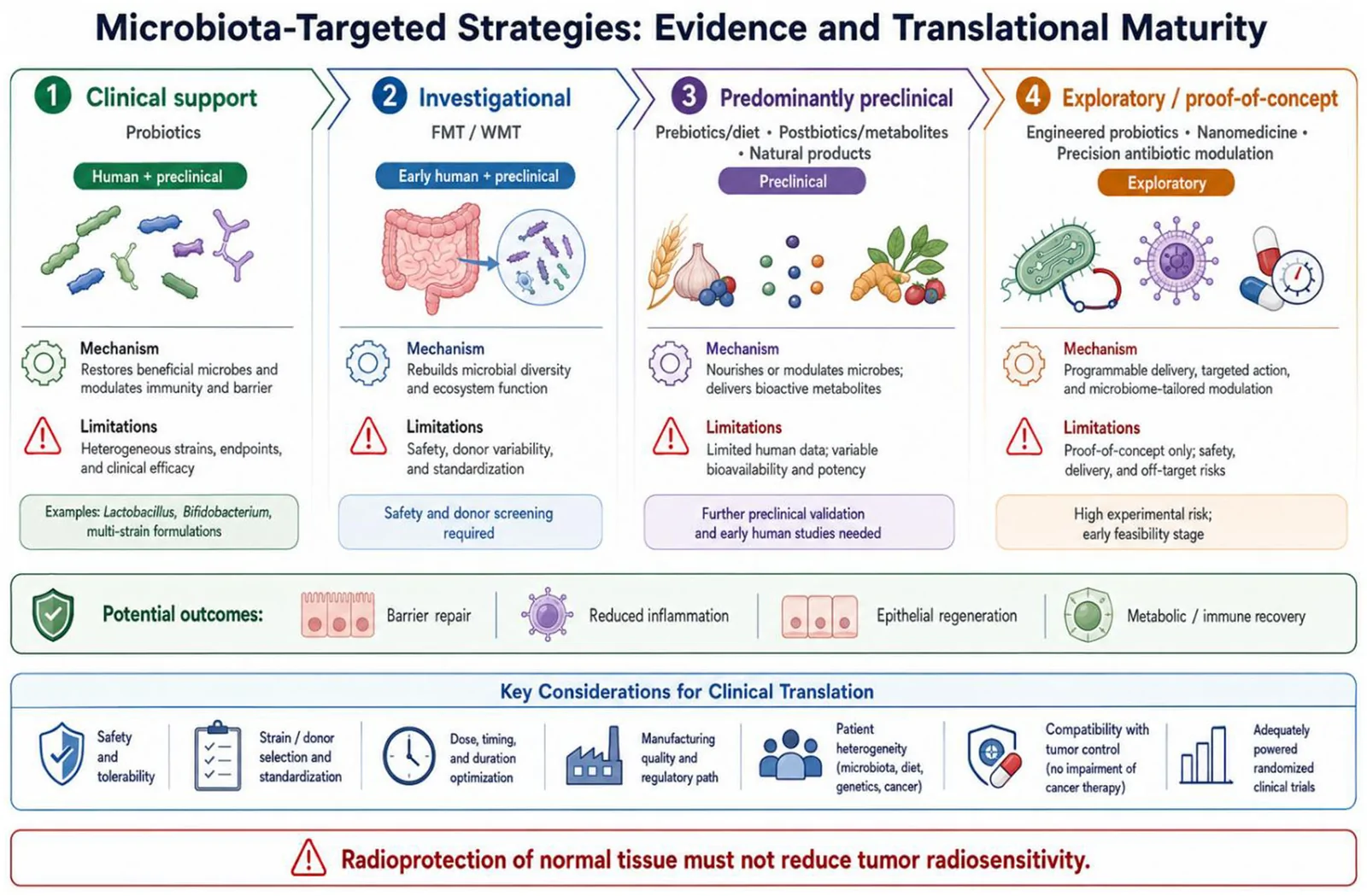
Evidence-based translational maturity of microbiota-targeted strategies for radiation-induced injury. Candidate interventions are organized according to their current evidence base and stage of clinical development. Probiotics have comparatively greater human interventional support, but their reported effects remain strain-, dose-, formulation-, endpoint-, and treatment-context dependent. Fecal microbiota transplantation (FMT) and washed microbiota transplantation (WMT) remain investigational, with preliminary human and preclinical evidence limited by donor variability, infectious risk, uncertain engraftment, manufacturing standardization, and regulatory complexity. Prebiotics and dietary interventions, postbiotics and defined microbial metabolites, and microbiota-modulating natural products remain predominantly preclinical in radiation injury. Engineered probiotics, microbiota-oriented nanomedicine, and precision antibiotic modulation are exploratory or proof-of-concept approaches. The potential outcomes illustrated—including barrier support, reduced inflammatory signaling, epithelial regeneration, and metabolic or immune recovery—are context dependent and should not be interpreted as equivalent across intervention classes. Clinical advancement will require standardized products and protocols, rigorous safety and biosafety assessment, optimized dose and timing, patient stratification, scalable manufacturing, adequately powered randomized controlled trials, and confirmation that normal-tissue protection does not compromise tumor radiosensitivity or anticancer efficacy.

Taken together, microbiota-directed strategies differ substantially in translational maturity. Probiotics currently have comparatively greater human interventional evidence, although their efficacy remains strain-, dose-, formulation-, and treatment-context dependent. Dietary and prebiotic approaches may offer practical advantages in safety and scalability, but radiation-specific human evidence remains limited and their effects are likely to be individualized. Defined postbiotics and microbial metabolites provide greater compositional control and mechanistic precision than live microbial communities but remain predominantly preclinical in radiation injury. FMT/WMT can reconstruct complex microbial ecosystems and has shown preliminary efficacy signals in radiation enteritis; however, donor variability, infection risk, regulatory complexity, uncertain engraftment, and limited controlled evidence preclude routine clinical implementation. Engineered probiotics and microbiota-oriented nanomedicine offer substantial therapeutic programmability but remain early-stage approaches requiring extensive biosafety, manufacturing, and regulatory validation. Therefore, therapeutic promise should not be equated with clinical readiness, and future development should prioritize interventions according to the combined criteria of efficacy, safety, reproducibility, scalability, regulatory feasibility, and compatibility with anticancer therapy. Figure 4 illustrates the translational continuum of these intervention categories, whereas Table 3 provides a comparative assessment of their current evidence, observed efficacy signals, safety considerations, scalability, and clinical readiness (Wang et al., 2024; Devaraj et al., 2019; Ding et al., 2020; Tao et al., 2026; Li et al., 2025; Wang et al., 2025; Zhang et al., 2025).

**Table 3:** Comparative evidence, observed efficacy signals, and translational readiness of microbiota-targeted strategies for radiation-induced injury.

Strategy	Current evidence base	Observed efficacy signal	Principal safety considerations	Standardization and scalability	Current translational stage
Probiotics	Controlled human trials, meta-analyses, and strain-specific animal studies	Human efficacy signal; heterogeneous	Generally favorable, but caution is required in patients with severe immunosuppression, neutropenia, or major barrier disruption	Relatively scalable, but strain identity, viability, dose, formulation, and batch consistency require standardization	Comparatively most advanced, but not established as standardized therapy
Prebiotics and dietary interventions	Predominantly preclinical radiation studies; limited radiation-specific human evidence	Preclinical efficacy signal	Generally favorable, but tolerability, nutritional status, gastrointestinal symptoms, and individualized metabolic responses require consideration	Highly scalable in principle; effects depend on formulation, dietary background, adherence, and baseline microbiota	Predominantly preclinical for radiation-specific applications
Postbiotics and defined microbial metabolites	Mainly animal, cellular, and organoid studies involving selected defined metabolites	Preclinical efficacy signal	Compound-, dose-, route-, and tissue-dependent; off-target metabolic and receptor-mediated effects require evaluation	High compositional controllability, but pharmacokinetics, stability, formulation, and dosing remain unresolved	Predominantly preclinical
FMT/WMT	Community-transfer animal studies and limited early human investigations	Preliminary human + preclinical signal	Transmission of infectious or multidrug-resistant organisms, donor-derived traits, invasive infection, and uncertain long-term ecological effects	Limited by donor variability, pathogen screening, manufacturing procedures, storage, engraftment variability, and jurisdiction-specific regulation	Investigational
Antibiotic modulation	Context-dependent preclinical and supportive clinical evidence	Context-dependent or uncertain	Loss of beneficial microbial functions, antimicrobial resistance, opportunistic infection, and prolonged dysbiosis	Drug manufacture is standardized, but biological effects depend on spectrum, dose, timing, baseline microbiota, and host immune status	Supportive or experimental conditioning strategy
Natural products	Mainly preclinical radiation studies; selected clinical studies may address radiation toxicity without establishing microbiota dependence	Preclinical efficacy signal; microbiota dependence often unconfirmed	Compound-, dose-, formulation-, and interaction-dependent; potential herb–drug interactions require evaluation	Moderate; limited by compositional heterogeneity, bioavailability, microbial biotransformation, and batch variability	Predominantly preclinical for microbiota-mediated applications
Engineered probiotics	Preclinical proof-of-concept studies	Preclinical efficacy signal	Genetic stability, biocontainment, horizontal gene transfer, environmental release, dose controllability, and host immune responses	Potentially scalable, but manufacturing, genetic quality control, storage, and regulation are complex	Exploratory/proof-of-concept
Nanomedicine-assisted microbiota/metabolite therapy	Early preclinical delivery-platform and formulation studies	Preclinical efficacy signal	Material-dependent toxicity, biodistribution, biodegradation, mucosal effects, immune interactions, and long-term ecological consequences	Limited by formulation complexity, reproducibility, scale-up, quality control, and regulatory requirements	Exploratory/proof-of-concept

“Human efficacy signal; heterogeneous” indicates that at least one controlled human study has reported a beneficial clinical signal, but substantial heterogeneity remains across strains, formulations, doses, endpoints, or study designs. “Preliminary human + preclinical signal” refers to small, early-stage, or uncontrolled human investigations supported by experimental studies but lacking confirmatory randomized evidence. “Preclinical efficacy signal” indicates that evidence is derived primarily from animal, cellular, or organoid models. “Context-dependent or uncertain” indicates that the direction or magnitude of benefit varies according to the intervention, timing, host condition, or baseline microbiota. These descriptors represent a narrative comparative assessment and do not constitute a formal GRADE evaluation. FMT, fecal microbiota transplantation; GRADE, Grading of Recommendations Assessment, Development and Evaluation; WMT, washed microbiota transplantation.

### 6.1 Probiotics

Among microbiota-directed interventions, probiotics currently have comparatively greater human clinical evidence for radiation-associated gastrointestinal toxicity. Randomized studies and meta-analyses suggest that selected probiotic formulations may reduce the incidence or severity of radiation-induced diarrhea, although substantial heterogeneity in bacterial strains, dose, treatment duration, radiotherapy regimens, and clinical endpoints limits definitive conclusions (Devaraj et al., 2019; Delia et al., 2007). More recent clinical investigations, including a phase II trial of *Bifidobacterium longum* subsp. *longum* BL21 in patients undergoing pelvic radiotherapy, further support continued clinical evaluation of strain-specific probiotic strategies (Yang et al., 2025).

Preclinical studies provide more detailed mechanistic evidence for selected strains such as Lactobacillus rhamnosus GG (LGG), but these mechanisms should not be generalized to all probiotics. Likewise, evidence supporting multi-strain consortia and next-generation candidates such as Akkermansia muciniphila is largely preclinical. Thus, probiotics represent the microbiota-based approach with the most developed clinical evidence in this review, but they cannot yet be considered standardized radioprotective therapy. Major barriers include strain-specific efficacy, manufacturing consistency, colonization variability, safety in severely immunocompromised patients, and the need to confirm that normal-tissue protection does not compromise tumor response (Ciorba et al., 2012; Riehl et al., 2019; Jian et al., 2022; Zhang et al., 2024; Xia et al., 2021; Delia et al., 2007; Yang et al., 2025).

### 6.2 Prebiotics and dietary interventions

Prebiotic and dietary interventions remain predominantly preclinical in radiation injury, although their practical safety and scalability make them attractive candidates for future translation. Experimental studies indicate that fermentable fiber and inulin can alter microbial metabolic output and reduce intestinal toxicity after irradiation. Importantly, these interventions should be interpreted as ecological manipulations rather than as direct proof that a specific microbial metabolite mediates protection (Garcia-Peris et al., 2016; Then et al., 2024, 2020; Liu et al., 2023; Eaton et al., 2022).

Recent work provides a more mechanistically informative example. Li et al. reported that phytate promoted intestinal colonization by Parasutterella and alleviated hematopoietic and intestinal radiation injury in experimental models; microbial remodeling was accompanied by altered production of functional metabolites, including 3-phenyllactic acid and N-acetyl-L-leucine (Li et al., 2025). Because this study combined dietary intervention with microbial and metabolic analyses, it strengthens the diet–microbiota–host hypothesis but remains preclinical. Whether phytate supplementation or Parasutterella-directed manipulation benefits patients receiving radiotherapy has not been established. Accordingly, dietary and prebiotic approaches should currently be regarded as low-complexity, predominantly preclinical microbiota-modulating strategies. Future clinical studies should define dose, dietary background, treatment timing, microbiome-dependent responders, nutritional safety, and effects on tumor control.

### 6.3 Postbiotics and microbial metabolites

Defined microbial metabolites offer conceptual advantages over live microorganisms because their composition and dose can be controlled more precisely. However, in radiation injury, the clinical evidence supporting postbiotic or purified-metabolite therapy remains very limited. SCFAs, selected tryptophan metabolites, 3HB, and LCA have undergone functional testing in experimental models, but most have not entered controlled clinical evaluation for radioprotection (Guo et al., 2020; Xie et al., 2024; Vernia et al., 2000; Zhang et al., 2021; He et al., 2023; Ouyang et al., 2026; Ge et al., 2024).

Therefore, postbiotics should be regarded as mechanistically attractive but predominantly preclinical therapeutic candidates. Their translation will require characterization of pharmacokinetics, optimal exposure, tissue distribution, potential off-target effects, formulation stability, and possible effects on tumor radiosensitivity. Moreover, a single metabolite is unlikely to reproduce the complex functional output of an intact microbial ecosystem (Koh et al., 2016; Xie et al., 2024; Ouyang et al., 2026; Ge et al., 2024; Vernia et al., 2000; Zhang et al., 2021).

### 6.4 Fecal microbiota transplantation

FMT provides a more global strategy for microbial ecosystem reconstruction than strain- or metabolite-based interventions. Animal studies support the ability of FMT to transfer aspects of radiation-associated microbial phenotypes, providing stronger evidence for microbiota dependence than compositional profiling alone (Cui et al., 2017).

Human evidence, however, remains preliminary. Early studies in chronic radiation enteritis reported symptomatic and endoscopic improvement following FMT, supporting clinical proof-of-concept but not establishing efficacy (Ding et al., 2020). More recently, WMT has been investigated in radiation enteritis, and Holdemanella biformis has been identified as a candidate organism capable of augmenting WMT efficacy, with functional validation extending into experimental models (Wang et al., 2026). Despite these encouraging findings, FMT remains an investigational strategy for radiation-induced injury and presents important safety and regulatory challenges. These concerns are particularly relevant in patients with cancer, who may have radiation-induced mucosal barrier disruption, neutropenia, chemotherapy-associated immunosuppression, or other conditions that increase susceptibility to invasive infection. Transmission of multidrug-resistant organisms through FMT has resulted in severe infections and death in immunocompromised recipients in other clinical settings (DeFilipp et al., 2019), emphasizing the necessity for rigorous donor screening and pathogen surveillance. Screening protocols must therefore account not only for conventional enteric pathogens but also for multidrug-resistant organisms, emerging infectious agents, and potentially transmissible components of the donor microbiome.

Donor-dependent variability represents an additional obstacle. Microbial composition, functional gene repertoires, metabolite production, bacteriophages, and engraftment efficiency differ substantially among donors, potentially resulting in heterogeneous therapeutic responses. Recipient factors—including baseline microbiota, diet, antibiotic exposure, immune status, and ongoing anticancer therapy—may further determine engraftment and clinical efficacy. Moreover, the long-term consequences of transferring a complex microbial ecosystem remain incompletely understood, including the theoretical transmission of microbial traits associated with metabolic, inflammatory, or other chronic diseases (Cammarota et al., 2017).

Regulatory frameworks for FMT also differ among jurisdictions and remain more clearly established for recurrent Clostridioides difficile infection than for radiation enteritis. Therefore, FMT for radiation-induced injury should currently be regarded as investigational rather than established clinical therapy. Standardized donor qualification, manufacturing procedures, microbial and pathogen quality control, longitudinal safety surveillance, and prospective controlled trials will be required before broader implementation in radiation oncology. Defined microbial consortia or functionally characterized microbiota preparations may ultimately provide greater reproducibility and regulatory tractability than conventional donor-derived FMT (Yang et al., 2023; Khorashadizadeh et al., 2024; Wang et al., 2025; Tao et al., 2026; Li et al., 2025; DeFilipp et al., 2019; Cammarota et al., 2017).

### 6.5 Antibiotic modulation

Antibiotics should not be considered a direct microbiota-restorative radioprotective therapy. Preclinical studies indicate that selected regimens may reduce pathogen burden, bacterial translocation, or inflammatory injury under specific conditions, whereas broad-spectrum depletion can simultaneously remove beneficial microbial functions. The direction of effect therefore depends on drug spectrum, timing, baseline microbiota, host immune status, and radiation context (Yang et al., 2023; Khorashadizadeh et al., 2024; Wang et al., 2025; Tao et al., 2026; Li et al., 2025).

Consequently, antibiotic manipulation is best viewed as an experimental conditioning or supportive strategy rather than an established radioprotective intervention. Concepts such as selective decolonization followed by probiotics, postbiotics, or FMT are biologically plausible but remain insufficiently validated in clinical radiation settings (Yang et al., 2023; Khorashadizadeh et al., 2024; Wang et al., 2025; Tao et al., 2026; Li et al., 2025).

### 6.6 Natural products targeting gut microbiota and metabolites

Natural products represent a heterogeneous class of compounds with potential microbiota-modulating activity. Radiation-specific studies involving epigallocatechin-3-gallate (EGCG), berberine, polysaccharides, proanthocyanidins, and related compounds provide evidence of tissue protection in experimental settings, but in many studies the microbiota changes in parallel with direct antioxidant, anti-inflammatory, or regenerative effects. Therefore, microbiota modulation should not automatically be interpreted as the causal mechanism of radioprotection unless microbiota dependence is demonstrated by depletion, transfer, or reconstitution experiments (Liu et al., 2023; Li et al., 2010).

Although selected natural products have entered clinical investigation for radiation-associated toxicity, most microbiota-specific mechanistic claims remain preclinical. Their translation is further complicated by heterogeneous composition, variable bioavailability, microbial biotransformation, formulation differences, and batch standardization (Li et al., 2010).

### 6.7 Engineered probiotics and synthetic microbiome therapeutics: emerging preclinical platforms

Engineered probiotics provide a direct means of testing whether sustained local delivery of a defined microbial product can modify radiation injury. Gao et al. engineered *Escherichia coli* Nissle 1917 to produce propionate and demonstrated improved intestinal outcomes in a mouse model of abdominal irradiation (Gao et al., 2026). This represents strong proof-of-concept for a programmable living therapeutic but remains preclinical (Wang et al., 2025; Zhang et al., 2020; Mitra et al., 2025; Gao et al., 2026).

Accordingly, engineered probiotics should not be presented as near-clinical radioprotective modalities. Translation will require rigorous assessment of genetic stability, dose controllability, biocontainment, horizontal gene transfer, environmental release, manufacturing reproducibility, host immune interactions, and compatibility with anticancer radiotherapy (Gao et al., 2026; Wang et al., 2025; Zhang et al., 2020; Mitra et al., 2025).

### 6.8 Nanomedicine-assisted microbiota/metabolite therapy

Nanomedicine may improve the stability, localization, and controlled release of microbiota-associated metabolites or microbiota-modulating compounds. However, microbiota-oriented nanomedicine remains largely exploratory or at an early preclinical stage in radiation injury. Importantly, improved therapeutic efficacy of a nanoparticle formulation does not establish that its activity is mediated through the microbiota; many nanomaterials may exert direct antioxidant, anti-inflammatory, or pharmacokinetic effects independently of microbial remodeling (Zhang et al., 2025, 2022).

Future studies should therefore demonstrate microbiota dependence using depletion/reconstitution or comparable experimental approaches while simultaneously addressing biodistribution, biodegradation, mucosal toxicity, manufacturing reproducibility, long-term ecological effects, regulatory complexity, and potential effects on tumor radiosensitivity (Zhang et al., 2025, 2022).

## 7 Conclusions and future perspectives

Accumulating evidence implicates the gut microbiota–metabolite axis as an important modulator of radiation responses, but the strength of evidence varies substantially across microbial taxa, metabolites, host pathways, and experimental systems. Human observational studies have identified reproducible radiation-associated microbial and metabolic signatures, whereas direct causal evidence has been established only for selected microorganisms, metabolites, and signaling pathways in preclinical models. These distinctions are critical: association with radiation tolerance or toxicity does not by itself demonstrate that a microbial feature is necessary or sufficient for the phenotype (Blake et al., 2024; Wang et al., 2024; Guo et al., 2020; Iacovacci et al., 2024; Ma et al., 2025; Xie et al., 2024; Ouyang et al., 2026; Ge et al., 2024).

The field should therefore move beyond increasingly detailed descriptive microbiome profiling toward hypothesis-driven causal experimentation. Priority approaches include germ-free and gnotobiotic models, antibiotic depletion followed by defined reconstitution, strain-level colonization, microbial consortia, metabolite supplementation and inhibition, receptor or pathway knockout, and human organoid systems. Integration of these approaches with metagenomics, metabolomics, lipidomics, transcriptomics, and spatial or single-cell technologies should enable the field to distinguish biomarkers from functional mediators (Blake et al., 2024; Wang et al., 2024; Guo et al., 2020; Sumner et al., 2007).

Therapeutically, microbiota-based strategies span markedly different levels of maturity. Probiotics currently have comparatively greater human clinical support, whereas FMT/WMT remains investigational and most defined metabolites, engineered bacteria, and microbiota-oriented nanomedicines remain preclinical or exploratory. Future translation will require not only demonstration of normal-tissue protection but also rigorous assessment of microbial safety, manufacturing reproducibility, patient selection, treatment timing, regulatory feasibility, and—critically—preservation of antitumor radiotherapy efficacy (Wang et al., 2024; Devaraj et al., 2019; Yang et al., 2025; Ding et al., 2020; Wang et al., 2026; Tao et al., 2026; Li et al., 2025; Wang et al., 2025; Zhang et al., 2025).

Among emerging areas, the microbiota–lipid metabolism–ferroptosis relationship is particularly hypothesis-generating. Selected metabolite pathways such as LCA-mediated ferroptosis regulation now have direct mechanistic support, but the broader axis remains incompletely defined. Likewise, baseline microbiome profiles may eventually contribute to toxicity-risk stratification, but predictive utility requires prospective validation across independent clinical cohorts (Iacovacci et al., 2024; Ma et al., 2025; Ouyang et al., 2026).

Overall, the most important transition for the field is not simply from conventional to more technologically sophisticated microbiota interventions, but from association to causation and from preclinical efficacy to evidence-based clinical translation. A rigorously validated understanding of the gut microbiota–metabolite axis may ultimately enable microbiome-informed strategies that reduce normal-tissue toxicity without compromising tumor control and thereby contribute to more precise radiation medicine.
